# N-Heteroacenes as an Organic Gain Medium for Room-Temperature Masers

**DOI:** 10.1021/acs.chemmater.3c00640

**Published:** 2023-05-23

**Authors:** Max Attwood, Xiaotian Xu, Michael Newns, Zhu Meng, Rebecca A. Ingle, Hao Wu, Xi Chen, Weidong Xu, Wern Ng, Temitope T. Abiola, Vasilios G. Stavros, Mark Oxborrow

**Affiliations:** † Department of Materials, Imperial College London, South Kensington Campus, Exhibition Road, London SW7 2AZ, U.K.; ‡ Molecular Sciences Research Hub, Department of Chemistry, Imperial College London, White City Campus, 82 Wood Lane, London W12 0BZ, U.K.; § Department of Chemistry, University College London, 20 Gordon Street, London WC1H 0AJ, U.K.; ∥ Center for Quantum Technology Research and Key Laboratory of Advanced Optoelectronic Quantum Architecture and Measurements, School of Physics, Beijing Institute of Technology, Beijing 100081, China; ⊥ Department of Chemistry, University of Toronto, 80 St. George Street, Toronto M5S 3H6, Canada; # Department of Chemistry, University of Warwick, Coventry CV4 7AL, U.K.; ∇ Department of Computer Science, University of Southern California, Los Angeles, California 90089, United States

## Abstract

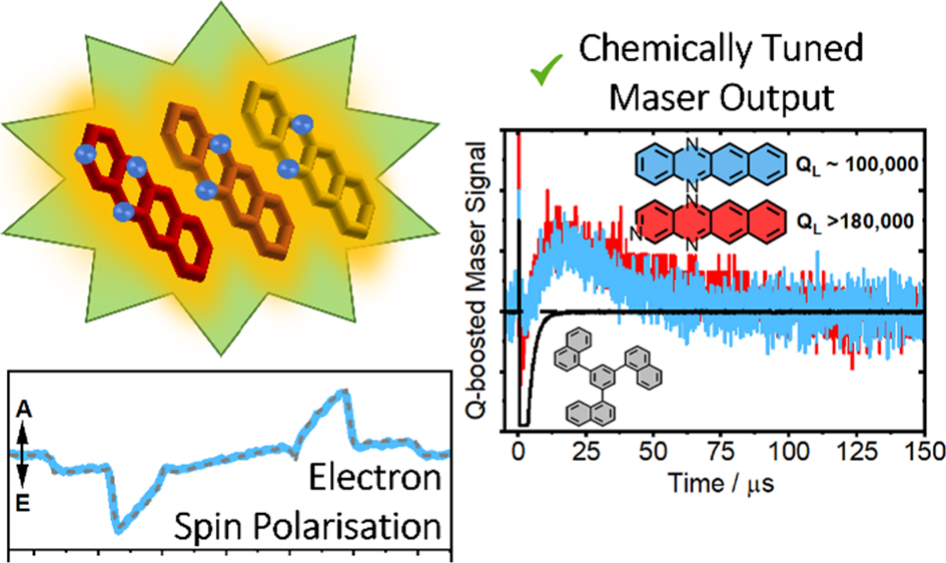

The development of future quantum devices such as the maser, i.e., the microwave analog of the laser, could be well-served by the exploration of chemically tunable organic materials. Current iterations of room-temperature organic solid-state masers are composed of an inert host material that is doped with a spin-active molecule. In this work, we systematically modulated the structure of three nitrogen-substituted tetracene derivatives to augment their photoexcited spin dynamics and then evaluated their potential as novel maser gain media by optical, computational, and electronic paramagnetic resonance (EPR) spectroscopy. To facilitate these investigations, we adopted an organic glass former, 1,3,5-tri(1-naphthyl)benzene to act as a universal host. These chemical modifications impacted the rates of intersystem crossing, triplet spin polarization, triplet decay, and spin–lattice relaxation, leading to significant consequences on the conditions required to surpass the maser threshold.

## 1 Introduction

Organic solid-state masers are an emerging class of ultralow noise amplifiers for microwave signals.^1−3^ These devices function by exploiting highly spin-polarized triplet states that are formed by spin-selective intersystem crossing (ISC). Interactions between magnetic dipoles of triplet electrons generate a zero-applied magnetic field splitting (ZFS) of spin sublevels (labeled T_*x*_, T_*y*_, and T_*z*_) characterized by axial |D| and rhombic |E| splitting components. The resonant microwave frequencies correspond to the transition energies between these triplet sublevels and typically correspond to Δ*E*_T*x*-T*z*_ = |D| + |E|, Δ*E*_T*y*–T*z*_ = |D| – |E|, and Δ*E*_Tx–Ty_ = 2|E|. The objective of current maser research is the development of materials with a high ISC yield (Φ_T_) coupled with a strong electron spin polarization favoring the higher spin sublevels.

For the archetypal organic maser gain material, pentacene-doped *p*-terphenyl (**Pc**:PTP), the triplet state populations (P_*x*_:P_*y*_:P_*z*_) are 0.76:0.16:0.08, and hence, the most strongly polarized T_*x*_–T_*z*_ transition has been used for masing.^4,5^ Innovations in resonator technology have reduced the size of zero-applied field (ZF) maser devices significantly and dulcified the requisite operating conditions,^3^ however, high signal-to-noise amplification still requires a powerful light source comprising either a Q-switched yellow laser or xenon flash lamp coupled with an invasive Ce:YAG luminescence concentrator.^7,8^ Even with these innovations, the demonstration of an organic maser operating as a continuous wave (CW) amplifier has remained elusive. Since masers are fundamentally limited by the electron spin dynamics of their gain media,^4,9^ further work is needed to design maser materials that can operate in miniaturized form and with moderate pumping sources.

Previously, semiempirical calculations of pentacene-like aromatic molecules have been used to evaluate their potential as maser gain media. The energy difference (Δ*E*_ST_) between the first excited singlet state (S_1_) and the most energetically proximal triplet state (T_2_) was used to predict the ISC rates (κ_ISC_).^10^ Subsequent *ab initio* investigations revealed that guest–host interactions significantly impact the relative energies of neighboring singlet and triplet states, exemplifying some of the inherent difficulty in computationally predicting maser materials.^11,12^ Nevertheless, these studies informed the discovery of 6,13-diazapentacene-doped *p*-terphenyl (**DAP**:PTP) as an alternative maser gain media operating with a 620 nm pulsed laser.^13^ The discovery of new maser candidates is also hindered by reliance on doped crystalline materials such as *p*-terphenyl. Crystalline hosts are highly selective toward their compatible dopants which limits the number of candidates that can be readily explored. Furthermore, the introduction of intricate invasive pump architectures is often not compatible with crystal growth methods such as Bridgman growth.^14^ Ideally, a host matrix should accomodate high concentrations of spin-active molecules, while also being compatible with multiple methods of optical pumping and form highly crystalline domains, minimizing inhomogeneous broadening associated with the triplet sublevels.

**Scheme 1:**
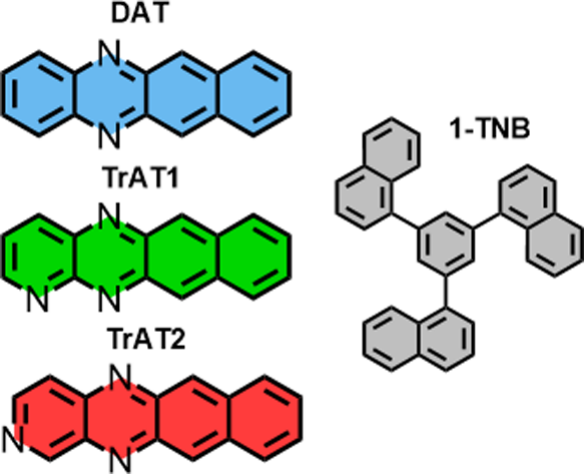
Structures of 1,3,5-Tri(1-naphthyl)benzene (1-TNB) Host Molecule and *N*-Substituted Tetracene Derivatives

In this work, we employ a universal host material, namely, 1-TNB, which forms an optically transmitting glass following a cycle of rapid heating and cooling.^15−17^ To exploit this material as a maser host and explore potential new maser gain materials, we have synthesized a series of nitrogen-substituted tetracene derivatives to modulate their optical activity (Scheme 1). The molecule **DAT** has previously been used as a source of spin-polarization for dynamic nuclear polarization^18−20^ and otherwise considered in organic optoelectronic applications.^21^ To modulate the photoexcited spin dynamics of this molecule, further nitrogen substitution was used to generate 1,5,12-triazatetracene (**TrAT1**) and 2,5,12-triazatetracene (**TrAT2**). Our analysis demonstrated that the optical band gap was modulated by the degree and position of nitrogen substitution, leading to noticeable differences in triplet formation in both dilute solutions and 1-TNB solid-state hosts. Interestingly, while the ZFS energies were similar between molecules, the triplet state populations varied significantly despite similar molecular architectures. Pulsed Q-boosted maser signals were subsequently recorded for the most favorable candidates.

## 2 Results

### 2.1 Steady-State Optical Spectroscopy

**Figure 1:**
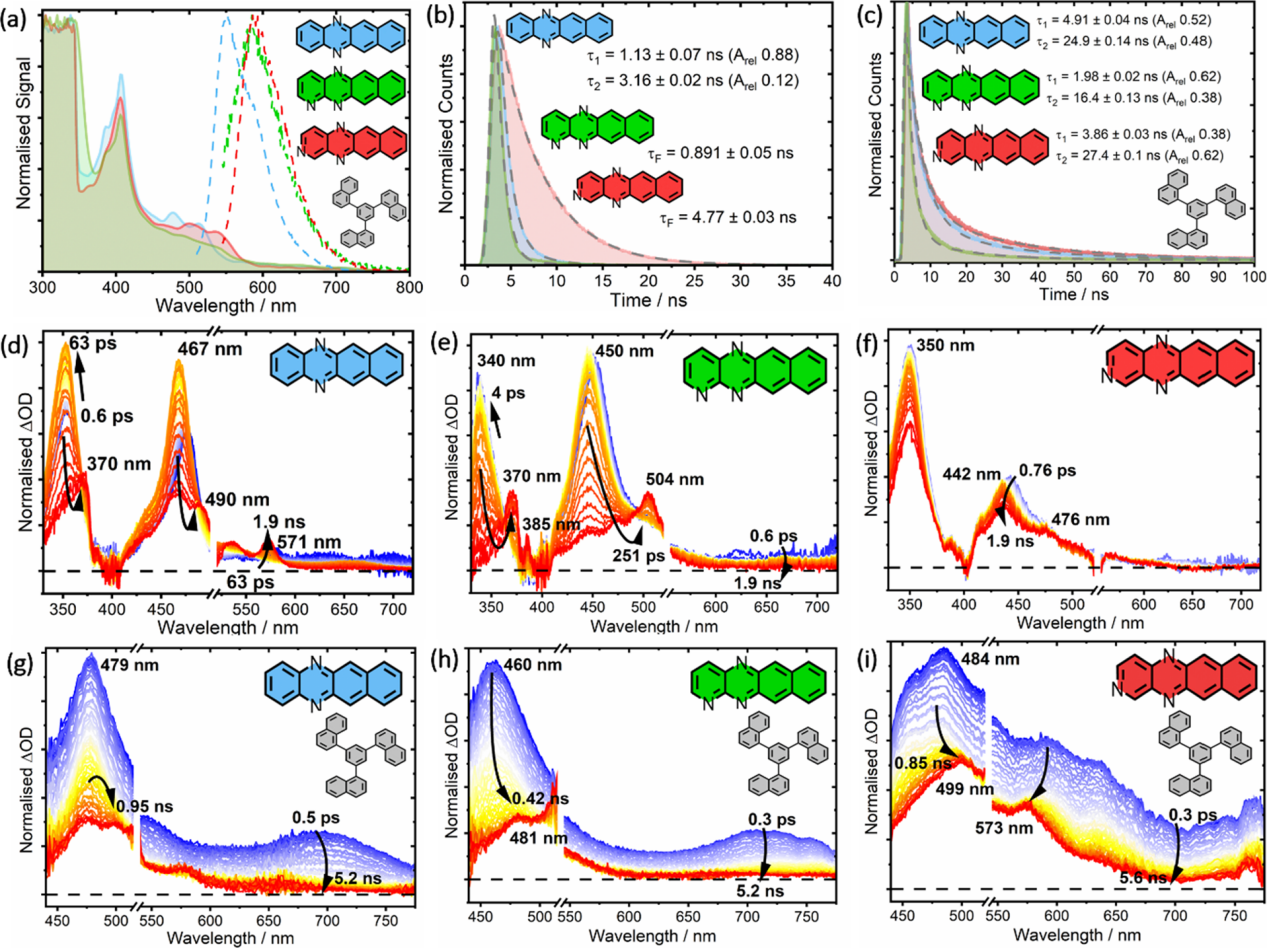
(a) Absorption (solid lines) and luminescence (dashed lines) spectroscopy of **DAT**, **TrAT1**, and **TrAT2**-doped 1-TNB (conc. 0.1% mol/mol). Time-correlated single photon counting (TCSPC) spectroscopy of **DAT**, **TrAT1**, and **TrAT2** in (b) THF solution or (c) doped in 1-TNB, with representing fits. (d–f) Femtosecond transient absorption spectroscopy (fsTAS) of **DAT**, **TrAT1**, and **TrAT2** in THF solution, respectively, and fsTAS (g–i) in **1-TNB** solid-state host. Samples were excited with 530 nm light except for DAT in THF which was excited at 510 nm. Sample concentrations in THF were set between 1.6 and 2 mM and 0.1% mol/mol in **1-TNB**.

1-TNB melts at 186 °C and can be cooled in air to form a stable pellet of amorphous glass.^15−17^ The rapid cooling ensures that candidate molecules remain trapped without aggregation, thereby yielding a sample with diluted spins that are not subject to bimolecular photophysical processes such as singlet fission or triplet–triplet annihilation. To understand how their incorporation into a 1-TNB host affects their photophysical properties, we conducted steady-state UV/Vis and fluorescence spectroscopy on tetrahydrofuran (THF) solutions of **DAT**, **TrAT1**, and **TrAT2** and doped 1-TNB samples (Figures 1a and S1a). When dissolved in THF, two prominent bands are observed with absorbance bands centered at 405 nm and ca. 510 nm for **DAT** and 530 nm for **TrAT1**/**TrAT2** (Figure S1a). Excitation with green light results in relatively bright fluorescence that peaks at 540 and 585 nm for **DAT** and **TrAT1**/**TrAT2**, respectively. When doped into a 1-TNB host at 0.1% mol/mol concentration, the main absorption and emission features are red-shifted by approximately 10 nm, peaking at 550 and 600 nm. Interestingly, irradiation at 400 nm yields anti-Kasha’s additional luminescence bands at 450 nm, as previously reported for THF solutions (Figure S1b).^21^ This is particularly evident for **TrAT1** where the 450 nm emission dominates the steady-state fluorescence spectrum. However, since 1-TNB is also excited by 405 nm light, this pump wavelength is unlikely to be optimal for downstream maser devices.

### 2.2 Transient Optical Spectroscopy

To begin probing the excited state dynamics of these materials, we measured their responses by TCSPC spectroscopy to determine their fluorescence lifetimes (τ_F_) in THF solution and doped 1-TNB systems (Figure 1b,c). The fluorescence decay curves were fitted using mono- or biexponential lifetimes, showing no significant improvement with additional exponential fitting terms (Figures S2a–c and S3a–c). These data revealed that the emission lifetime of doped 1-TNB systems was significantly slowed compared to THF solutions. **TrAT1** exhibited the shortest lifetime in both THF solution and 1-TNB with τ_F_ = 0.891 ns and a weight-averaged τ_F_ (τ_F,av_) of 7.46 ns, respectively. Similarly, **DAT** dissolved in THF returned a relatively fast τ_F,av_ of 1.31 ns, and a longer decay time of 14.5 ns in 1-TNB. The longest decay times of τ_F_ = 4.77 ns and τ_F,av_ = 18.5 ns were found for **TrAT2** in THF and 1-TNB, respectively. In each case, the dominant decay term for doped 1-TNB samples was similar in magnitude to dominant terms in THF, with the subsequent term ranging between 16.4 and 27.4 ns.

Since the detected transient fluorescence is associated with the absolute rate decay of S_1_ (κ_F_; κ_F_ = κ_rad_ + κ_IC_ + κ_ISC_), we sought to deconvolute contributions of fluorescence (κ_rad_), nonradiative internal conversion (κ_IC_), and ISC (κ_ISC_). Therefore, solutions of THF and doped 1-TNB samples were measured by fsTAS following excitation with green light to monitor the excited state dynamics. In THF solution, two excited state absorption (ESA) bands are initially observed at ∼350 and ∼450 nm for all three materials (Figure 1d–f). These features can be attributed to S_1_ → S_*n*_ electronic transitions, appearing at similar wavelengths to equivalent processes in tetracene^22,23^ and pentacene-derivatives.^24,25^ This association is supported by their similar S_1_ decay time determined by TCSPC spectroscopy. For **TrAT2**, the decay of S_1_ extends beyond the time window of our experiment (1.9 ns) making kinetic analysis impossible. For all three materials, the ESA bands reduce in intensity after 100 ps followed by the appearance of shoulder peaks which are most clearly observed for **DAT** at 370, 490, 537, and 571 nm, and for **TrAT1** at 370, 385, and 504 nm. Since the growth of these bands is too slow for vibrational cooling, which typically occurs within a few picoseconds,^26,27^ and based on their persistent growth up to 1.9 ns, these features are attributed to the lowest triplet state (T_1_) following ISC from S_1_. The T_1_ assignment (over higher T_*n*_ states) can be asserted since IC between T_*n*_ → T_1_ in linear acenes is typically very rapid (<10 ps).^28,29^ Furthermore, such processes have been noted for tetracene at similar wavelengths,^22,30^ while for **DAT** an absorption at 580 nm was used to determine the triplet lifetime for **DAT**:PTP, which is close to the 571 nm band we observed.^18^ Since negative bands associated with a ground state bleach or stimulated emission are absent here, it is assumed that the observed spectra are also convoluted with an additional broad ESA which masks their contributions.

For the doped 1-TNB samples, the measured time window was increased to 5.2 ns (5.6 ns for **TrAT2**:1-TNB) following the more gradual S_1_ relaxation indicated by TCSPC spectroscopy, and the detection window was adjusted to 440 and 800 nm due to the change in instrumentation. Again, ESA bands were observed between 460 and 484 nm, coupled with an additional broad ESA covering the entire spectrum (Figure 1h,i). The decay of these features coincides with the emegence of weak bands on similar time scales to T_1_ → T_*n*_ absorptions in THF solutions, beginning at approximately 1 ns in **DAT**:1-TNB at 580 nm, and 450 ps in **TrAT1**:1-TNB at 481 nm. Notably, the absorption band at 580 nm for **DAT**:1-TNB matches that used by Kouno et al., to determine the triplet lifetime of 120 μs.^18^ For **TrAT2**:1-TNB, S_1_ relaxation manifests as a band shift from 484 to 499 nm with the later appearance of an additional weak band at 573 nm. These features persist to the end of the experiment supporting an assignment to the T_1_ state, however, an estimation for the onset of absorption is impossible due to the broad nature of these absorptions.

Due to the convoluted spectral components, kinetic analysis required global analysis (GA) following singular value decomposition. In each case, two or three principal components were required to fit the fsTAS data, each of which have lifetimes corresponding to either a mono- or biexponential decay, as with TCSPC spectroscopy (Figure S5). For THF samples, the first and second fitted components generally accounted for S_1_ → S_N_ ESA bands. The third component contains features consistent with the triplet absorption bands. Due to the incomplete excited state decay within the accessible time window of our experiments, the absolute rate values derived from our analysis are likely inaccurate, and for **TrAT2** a sensible kinetic analysis was again impossible. However, the magnitude of the rates coupled with the onset time of triplet absorption can be used to infer that **TrAT1** (onset 250 ps, est. τ_ISC_ = 531 ps) undergoes ISC slightly more rapidly than **DAT** (onset 400 ps, est. τ_ISC_ = 882 ps), followed distantly by **TrAT2**. A similar analysis for 1-TNB samples was also conducted, however, due to their weak absorption convoluted with an extremely broad ESA, lower signal-to-noise, and dominance of the laser scatter, the triplet absorptions were not clearly represented in the principal components (Figure S6a–f). Furthermore, due to the incomplete S_1_ relaxation captured in these spectra, the corresponding estimated component lifetimes extended beyond the 5.2 ns time window of the experiment. Nevertheless, it is clear from these data that like the rate of fluorescence, the apparent κ_ISC_ is significantly slowed for **DAT**:1-TNB, **TrAT1**:1-TNB, and **TrAT2**:1-TNB.

With the limited information available following fsTAS of doped 1-TNB samples, we sought to supplement this data by quantifying their fluorescence quantum yield (Φ_F_, see SI for details). Since the gain of a maser device is proportional to the number of polarized triplets generated by its spin-active media upon photoexcitation, it is expected that the better candidates would have a lower Φ_F_. Indeed, our measurements indicated that the Φ_F_ for **DAT**:1-TNB and **TrAT1**:1-TNB was 37.3 and 38.9%, respectively, while **TrAT2**:1-TNB exhibited Φ_F_ = 58.3% (Table 1). The larger Φ_F_ value for **TrAT2**:1-TNB is consistent with its extended S_1_ lifetime revealed by TCSPC and fsTAS, which is associated with less efficient ISC.

**Table 1:** Photophysical Parameters Derived from fsTAS and TCSPC

system	host	Φ_F_/%	τ_F_/ns	κ_rad_^a^/ns^–1^	κ_ISC_^b^/ns^–1^	κ_IC_/ns^–1^	Φ_IC_/%	Φ_T_^a^/%	τ_L_/μs
**pentacene**	PTP		∼9					62.5^34^	89^18^
**tetracene**	toluene^30,32^	16	4.2	0.038	0.147	0.053	22	62	0.63
	benzene^33^	15	5.2	0.029	0.131	0.03	17	68	
	anthracene^35^		18.4						
**DAT**	PTP^18^								120^18^
	THF		1.37 ± 0.05						
	1-TNB	37.3 ± 1.6	14.5 ± 0.09	0.026	0.043 (κ_nr_)				
**TrAT1**	THF		0.891 ± 0.05						
	1-TNB	38.9 ± 1.6	7.46 ± 0.06	0.052	0.082 (κ_nr_)				
**TrAT2**	THF		4.77 ± 0.03						
	1-TNB	58.3 ± 1.6	18.5 ± 0.07	0.032	0.022 (κ_nr_)				

a (31) κ_rad_ = Φ_F_/τ_F_; τ_F_ = (κ_rad_ + κ_nr_)^−1^; κ_nr_ = κ_ISC_ + κ_IC_.

b (36) κ_F_ = κ_rad_ + κ_IC_ + κ_ICS_; Φ_T_ = Φ_F_(κ_ICS_/κ_rad_).

For isolated molecules and in the absence of photochemical reactions, κ_F_ = κ_rad_ + κ_IC_ + κ_ISC_. Therefore, using the experimentally determined values for Φ_F_ and τ_F(av)_ (), κ_rad_ can be estimated by κ_rad_ = .^31^ This further permits the assignment of an upper limit on the overall nonradiative decay rate, κ_nr_ (= κ_IC_ + κ_ISC_ = κ_F_ – κ_rad_; Table 1), as 0.043, 0.082, and 0.022 ns^–1^ for **DAT**:1-TNB, **TrAT1**:1-TNB, and **TrAT2**:1-TNB, respectively. Interestingly, these values are similar to those for the values for tetracene in nonpolar aromatic solvents where Φ_F_ values between 10 and 16% were reported,^30,32,33^ indicating less efficient triplet formation in these azatetracene samples.

### 2.3 Density Functional Theory

**Figure 2:**
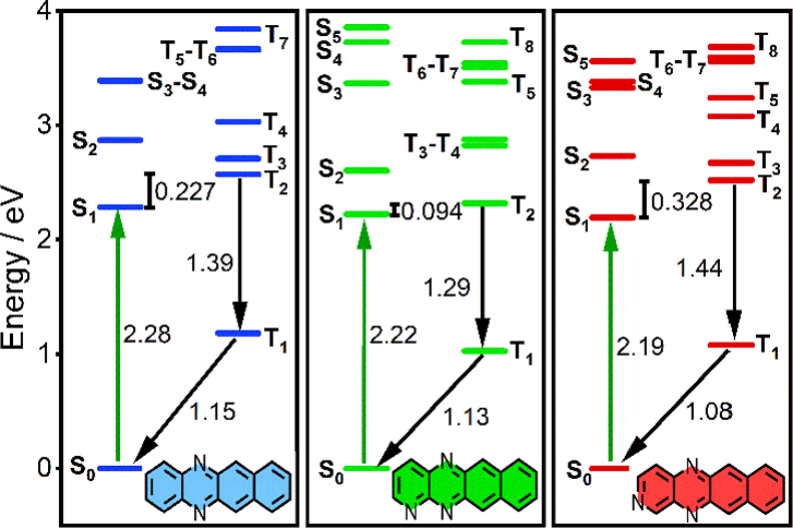
Electronic state Jablonski diagrams for **DAT**, **TrAT1**, and **TrAT2** estimated using TD-DFT calculations. Calculations were performed at the B3LYP 6-311G++(d,p) level using Gaussian09 software with an IEFPCM solvent model of benzene. Transition energies given in units of eV.

To further understand our observed optical properties, we performed density functional theory (DFT) calculations to help characterize the singlet and triplet state energies. Following the formalism described by Bogatko et al., for the computational evaluation of maser candidates,^10^ the S_0_ state energy was normalized to zero, and subsequent excited states were positioned according to optical band gaps as determined by time-dependent (TD)-DFT calculations (Figure 2, see SI for details). The energy of the ground state triplet (T_1_) was estimated using the energy difference between the S_0_ state and highest triplet singly occupied molecular orbital energy, defined as Δ*E*_TS_ (Table 2). The excited triplet states are thus found by adding Δ*E*_TS_ to the optical excitation energies for T_1_ → T_*n*_ transitions. This approach permits the examination of singlet and triplet state energy gaps (Δ*E*_ST_) and the construction of Jablonski diagrams.

**Table 2:** Singlet and Triplet Energy Differences Derived by TD-DFT Calculations (in Units of eV)

	S_0_–S_1_	Exp.^a^	Δ*E*_ST_ (S_1_–T_2_)	Δ*E*_TS_ (T_1_–S_0_)
**DAT**	2.28	2.45	0.227	1.15
**TrAT1**	2.22	2.40	0.094	1.13
**TrAT2**	2.19	2.32	0.328	1.08

a Determined from the leading edge λ_max_ according to UV–Vis spectroscopy.

The calculated optical excitation energies closely reproduced experimental trends observed by UV/Vis spectroscopy with only a ca. 7% difference in the absolute values (see Figure S7), typical for TD-DFT calculations. The band gaps were found to increase from **TrAT2** to **TrAT1** to **DAT**, with **TrAT1** estimated to exhibit the smallest Δ*E*_ST_ (= 0.094 eV) and **TrAT2** the largest (= 0.328 eV, Table 2). For small organic materials without high atomic weight species or functional groups that significantly mix singlet and triplet states via spin-orbit coupling,^37,38^ κ_ISC_ is often approximated to exponentially depend on the energy difference between the initial singlet state (S_1_) and the final triplet state (T_2_), according to the Fermi golden rule.^39,40^ To investigate whether this holds true for our nitrogen-substituted tetracene derivatives, quantum calculations were performed to estimate the spin-orbit coupling matrix elements between singlet and triplet states (Tables S5–S7). These data indicate relatively weak coupling values, particularly between S_1_ and T_*n*_ states, always less than 10 cm^–1^_._ By comparison, species with enhanced ISC due to species such as transition metals,^41,42^ sulfur,^43,44^ selenium,^45,46^ halogens, or nonbonding functional groups^37,38,47−49^ typically exhibit SOC parameters exceeding 100 cm^–1^. Interestingly, analysis of the natural transition orbitals reveals that the T_2_ state exhibits a clear nonbonding orbital character, particularly for **TrAT1** and **TrAT2** (Figure S8). According to El-Sayed rules,^50,51^ it might be expected that with a (π,π*)-type S_1_ state, **TrAT1**, and **TrAT2** would exhibit enhanced ISC compared to **DAT**, however, while **TrAT1** has been inferred to undergo slightly faster ISC, the slow S_1_ relaxation for **TrAT2** indicates that this is not the case. Therefore, here it is likely that ISC is still dominated by Δ*E*_ST_. This idea is supported by the larger Φ_F_ for **TrAT2**:1-TNB compared to **DAT**:1-TNB or **TrAT2**:1-TNB.

### 2.4 X-Band Time-Resolved Electronic Paramagnetic Resonance Spectroscopy

To further examine whether the triplet states of these materials are suitable as maser gain media, we also performed photoexcited X-band time-resolved electron paramagnetic resonance (trEPR) spectroscopy to determine their triplet sublevel populations and ZFS frequencies.

**Figure 3:**
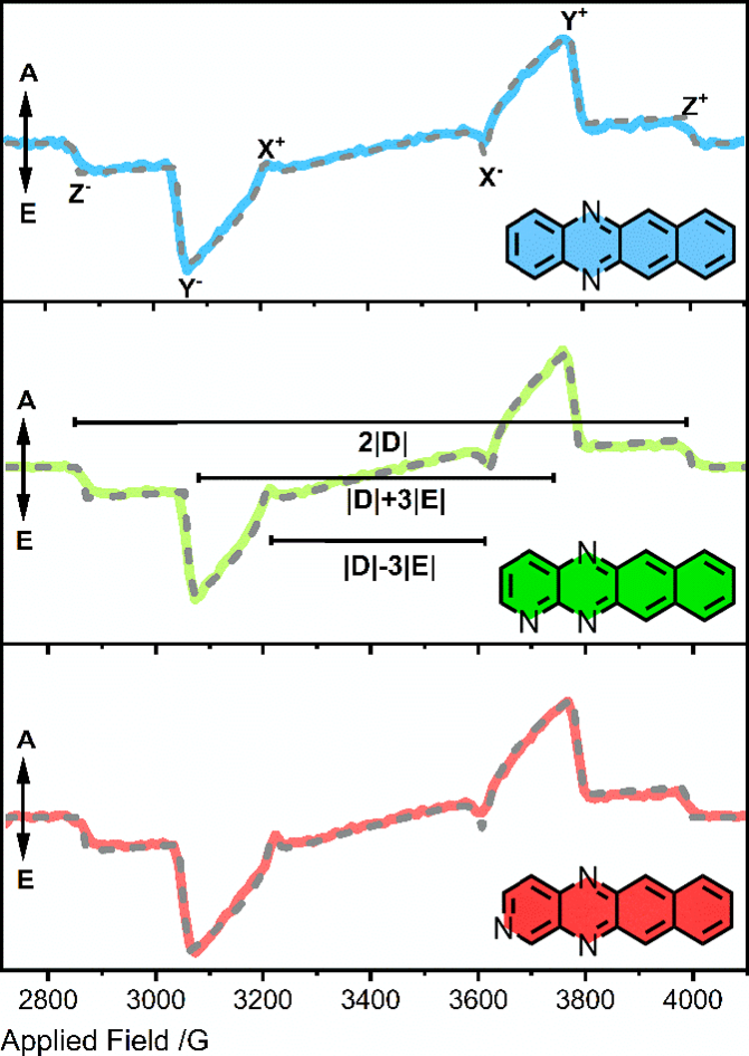
X-band photoexcited trEPR spectra for **DAT**:1-TNB (blue), **TrAT1**:1-TNB (green), and **TrAT2**:1-TNB (red). Traces are taken 400 ns after laser flash, with samples made to a concentration of 0.1% mol/mol and illuminated with 5–8 ns pulses of 532 nm light measured at 7 mJ/pulse. Gray dashed lines represent fittings performed using the EasySpin toolbox (see the SI for details).

As expected for amorphous glass samples, triplet signals consistent with the presence of powders were recorded for all materials. The transient signals were comprised of emissive (E) low-field and absorptive (A) high-field signals (Figure 3). The emissive phase for each material persists for ∼10 μs followed by a weak absorptive phase lasting up to 90 μs. To determine the triplet sublevel populations and ZFS, the signals were simulated using the Matlab EasySpin (v6.0.0) toolbox (see SI for details).^52^ All our materials were found to have positive T_*x*_–T_*z*_ and T_*x*_–T_*y*_ population differences (Table 3). It is notable that **DAT**:PTP exhibits a larger T_*z*_ population than **DAT**:1-TNB (0.18 vs 0.08) but a similar T_*x*_ population. Interestingly, the triplet polarization for **TrAT1**:1-TNB was the weakest, with a T_*x*_:T_*z*_ ratio of just 2.9, whilst **TrAT2**:1-TNB is the only material to present with an apparently enhanced spin polarization compared to **Pc**:PTP. As expected for such closely related chemical structures, the ZFS energies were also similar with |D| values between 1582 and 1609 MHz, and |E| values between 155 and 193 MHz, similar to tetracene and **DAT**:PTP.^18^ Overall, based on the triplet sublevel populations **DAT**:1-TNB and **TrAT2**:1-TNB would appear to be narrowly better maser candidates than **TrAT1**:1-TNB.

**Table 3:** Triplet Parameters Derived from X-Band trEPR Spectroscopy Experiments

	host system	2D spectral fitting				
		D	E	T_*x*_–T_*z*_ (T_*y*_–T_*z*_)	P_*z*_:P_*y*_:P_*x*_	P_*x*_/P_*z*_
		/MHz				
**pentacene**	*p*-terphenyl	1395^53^	53^53^	1449 (1341)^53^	0.08:0.16:0.76^4^	9.5
		1315^18^	50^18^			
	1-TNB^17^	1385^17^	50^17^	1435 (1334)^17^	0.19:0.35:0.46^17^	2.4
**tetracene**	*p*-terphenyl	1653^54^	112^54^	1765 (1541)^54^	0.00:0.41:0.59^54^	N/A
**DAT**	*p*-terphenyl^18^	1598^18^	153^18^	1751 (1443)^18^	0.18:0.11:0.71^18^	3.7
	1-TNB^a^	1609	156	1765 (1453)	0.08:0.21:0.71	8.9
**TrAT1**	1-TNB^a^	1582	193	1775 (1389)	0.19:0.26:0.55	2.9
**TrAT2**	1-TNB^a^	1609	155	1764 (1454)	0.01:0.33:0.66	66

a Data from this work.

### 2.5 Spin Dynamics—ZF trEPR Spectroscopy

With the ZFS and triplet sublevel populations known, we conducted ZF trEPR spectroscopy to individually analyze their T_*x*_–T_*z*_, T_*y*_–T_*z*_, and T_*x*_–T_*y*_ transitions. Measurements were collected using inductively coupled (IC) tank resonators fabricated to resonate with each transition frequency band (see the SI for details). To determine the frequency with the most intense signal, measurements were conducted in steps of 5 MHz in proximity to the estimated transition frequencies (Figure S10).

**Figure 4:**
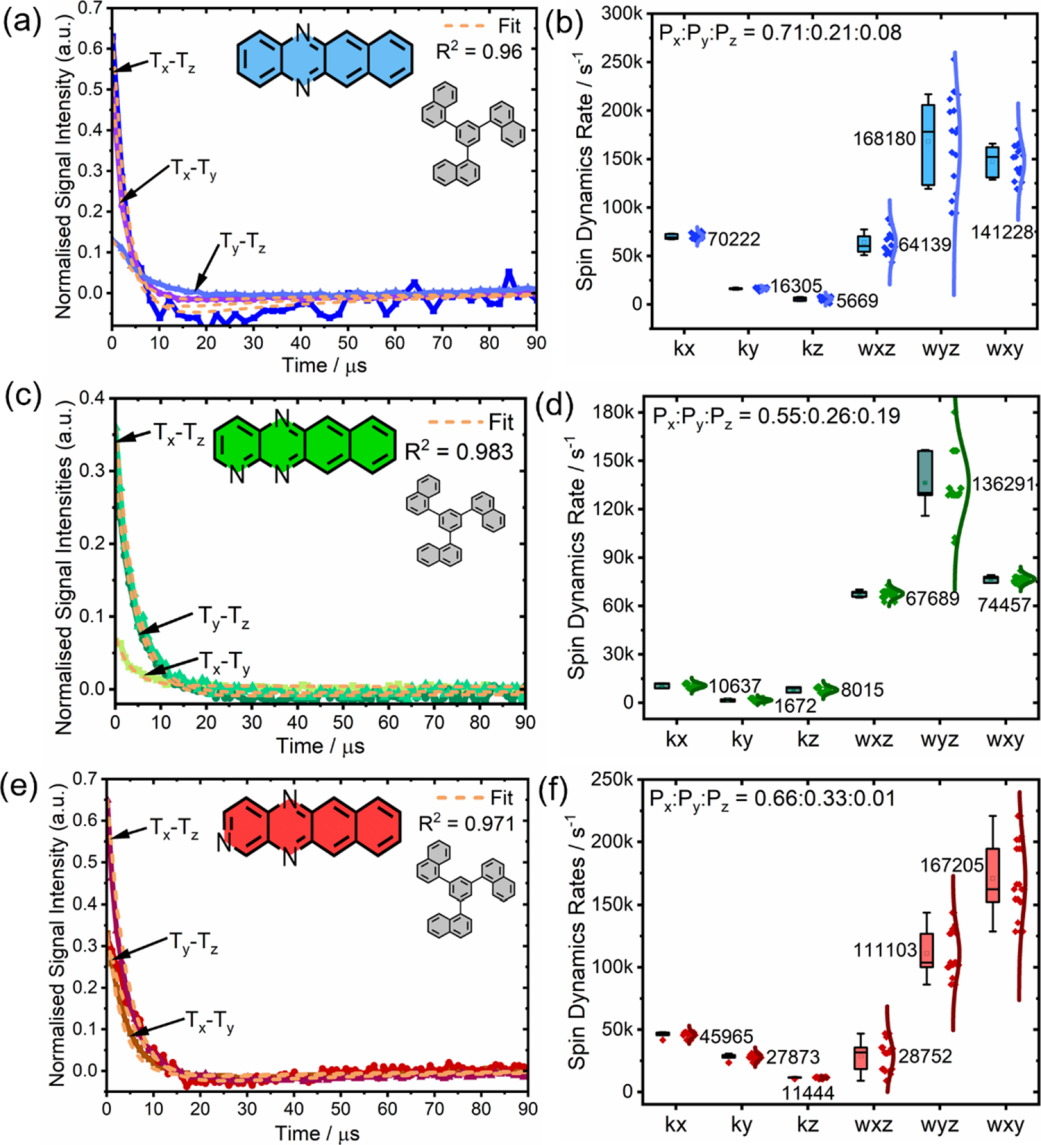
(a–c) ZF trEPR spectra of **DAT**:1-TNB (blue), **TrAT1**:1-TNB (green), and **TrAT2**:1-TNB (red); (d–f) transition-specific spin–lattice relaxation (ω_*ij*_) and sublevel-selective triplet decay rates (κ_*i*_) determined by fitting of ZF trEPR signals. Sample concentrations were 0.1% mol/mol and illuminated with 5–8 ns pulses of 510 or 532 nm light at 1 mJ/pulse. Individual signals were obtained at room temperature and averaged 512 times. Spin dynamics fittings are shown as orange dash lines.

Here, the most intense T_*x*_–T_*z*_ signal for **DAT**:1-TNB was observed at 1765 MHz, closely matching X-band-derived estimations, whereas optimal signals for **TrAT1**:1-TNB and **TrAT2**:1-TNB were found at 1755 MHz (Figure 4a–c). The transition linewidths were relatively broad with their full-width half-maximum exceeding 40 MHz, compared to approximately 3 MHz for a typical single crystal sample of **Pc**:PTP at a concentration of 0.1% mol/mol.^55^ As glass matrices do not exhibit any intrinsic long-range order, inhomogeneous broadening is expected to significantly increase compared to a crystal matrix due to different local magnetic environments. Since the transition linewidth is inversely proportional to the inhomogenous dephasing time (*T*_2_*) which is sensitive to magnetic inhomogeneity, the expected result is relatively broad ZF linewidths. Furthermore, hyperfine coupling to ^14^N nuclear spins results in additional broadening and peak asymmetry, as demonstrated by frequency sweep simulations using DFT-derived hyperfine coupling parameters for each molecule (Figure S9). As a result, the microwave power per unit frequency that is emitted by the sample during masing is significantly attenuated in glass matrices.

To further understand how these ZF trEPR signals might relate to their maser potential, the most intense signals were used to simulate their spin dynamics using the following relationship:
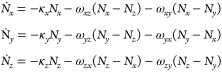
1Here, *N_i_* is the initial population of *i*-th sublevel as determined following X-band EPR spectroscopy; κ_*i*_ is the sublevel specific triplet decay rate to the singlet ground state, S_0_; ω_*ij*_ is the spin–lattice relaxation rate between the *i*-th and *j*-th sublevel. For this analysis, it was assumed that the *i*-*j* and *j*-*i* transition rates are equal at room temperature, as discussed previously.^4^ Fitted values correspond to R-squared values of >0.96, indicating that the fit represents 96% of the data (Table 4).

**Table 4:** Room-Temperature ZF trEPR Data and Fitted Spin Dynamics of the Triplet Ground State for Doped 1-TNB Samples^a^

sample	T_*x*_–T_*z*_	T_*y*_–T_*z*_	T_*x*_–T_*y*_	|D|	|E|	κ_*x*_	κ_*y*_	κ_*z*_	ω_*xz*_	ω_*yz*_	ω_*xy*_
**Pc:PTP**(4,7)	1450	1344	106	1397	53	2.8 ± 0.5	0.6 ± 0.2	0.2 ± 0.1	1.1 ± 0.2	2.2 ± 0.3	0.4 ± 0.2
						2.2	1.4	0.2	1.1	2.8	0.4
**DAT**:1-TNB	1765	1470	315	1608	158	7.43 ± 0.74	1.4 ± 0.04	0.74 ± 0.30	5.45 ± 2.0	16.9 ± 6.2	16.3 ± 2.9
**TrAT1**:1-TNB	1755	1375	325	1593	163	1.23 ± 0.23	0.83 ± 0.21	1.11 ± 0.25	7.02 ± 0.24	13.4 ± 0.77	5.39 ± 0.32
**TrAT2**:1-TNB	1755	1485	295	1608	148	4.17 ± 0.85	2.37 ± 0.61	1.04 ± 0.37	4.69 ± 2.03	8.62 ± 3.42	12.9 ± 4.56

a Units for κ_*i*_ and ω_*i*(*j*)_ values are given in 10^4^ s^-1^.

From these measurements, it was apparent that each compound has quite distinct spin dynamics (Figure 4). For **DAT**:1-TNB, the largest ω_*ij*_ values correspond to the T_*y*_–T_*z*_ and T_*x*_–T_*y*_ transitions, with T_*y*_ and T_*z*_ sublevels also exhibiting the smallest κ_*i*_ values. In practical terms, this means that relaxation from T_*y*_ and T_*x*_ states could lead to an accumulation of electrons in the T_*z*_ state. This contrasts with **TrAT1**:1-TNB, where ω_*xz*_ and ω_*xy*_ are smaller than ω_*yz*_ and coupled with a relatively small κ_*x*_ value compared to **DAT**:1-TNB, **TrAT2**:1-TNB, and even **Pc**:PTP. The realization of a short κ_*x*_, especially if coupled with a large κ_*z*_ parameter, would be an important advancement for the realization of a CW maser. Such a system would benefit from a reduced spontaneous loss of T_*x*_ polarization and more efficient recycling of spent T_*z*_ electrons. Interestingly, **TrAT2:**1-TNB essentially presents as a mix of **DAT**:1-TNB and **TrAT1**:1-TNB spin dynamics, with a relatively small ω_*xz*_ and relatively large *κ*_i_ values, where κ_*x*_ > κ_*y*_ > κ_*z*_. Overall, these azatetracene materials exhibit significantly larger ω_*ij*_ and κ_*i*_ values compared to **Pc**:PTP (Table 4), except for the κ_*x*_ value for **TrAT1**:1-TNB. This is consistent with the reduced electron spin polarization time at ZF.

To determine if these values are compatible with their use in a maser device, the spin dynamics can be related to the maser “cooperativity” (see eq 2). For a CW maser, the gain of individual materials can be summarized by the cooperativity factor adapted from ref (9), often denoted as η_maser_ (or C in ref (56)):
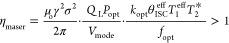
2Briefly, μ_o_ is the vacuum permittivity, γ is the reciprocal gyromagnetic ratio for maser transition (≡*–g*_J_μ_B_/*ℏ*), σ is the transition probability between the maser transition sublevels (assumed to be 0.5 at room temperature), *Q*_L_ is the loaded quality factor of the resonator, *P*_opt_ is the optical pump power, *V*_mode_ is the resonator’s magnetic mode volume, κ_opt_ is the absorption probability at the pump wavelength, *f*_opt_ is the optical pump frequency, θ_ISC_^eff^ is the effective ISC yield, *T*_2_* is the inhomogenous dephasing time, and finally *T*_1_^eff^ is the effective spin–lattice relaxation time, which acts as a ratio of the population-weighted spin dynamics.^7,9^ Maser oscillation with high signal-to-noise requires η_maser_ ≫ 1. The cooperativity thus depends on three “bulk” quantities comprised of a set of universal constants (μ_o_γ^2^σ^2^/2π), resonator-dependent parameters (*Q*_L_*P*_opt_/*V*_mode_), and finally a set of material-dependent parameters (*k*_opt_θ_ISC_^eff^*T*_1_^eff^*T*_2_^*^/*f*_opt_).

While materials with η_maser_ values close to unity are practically difficult to measure, in principle, the ability to CW mase ultimately depends on *T*_1_^eff^—the only parameter with a potentially negative sign (described in ref (9)). The sign of *T*_1_^eff^ is determined by its numerator which can be used as a figure of merit concerning the triplet spin dynamics, denoted as κ_rates_:^7^
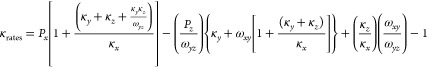
3

This term can be straightforwardly determined using the values reported in Table 4, yielding values of −0.06 ± 0.95 for **DAT**:1-TNB, 0.63 ± 0.51 for **TrAT1:**1-TNB, and 0.66 ± 0.86 for **TrAT2:**1-TNB. Therefore, based on this analysis all three azatetracene-doped 1-TNB samples may have the potential to CW mase within experimental error. It is interesting to note the sensitivity of κ_rates_ to errors in κ_*i*_ and ω_*ij*_ values. Surprisingly, despite the relatively obtuse nature of the **TrAT1**:1-TNB triplet decay compared to **DAT**:1-TNB and **TrAT2**:1-TNB, κ_rates_ is close to **TrAT2**:1-TNB which has a smaller ω_*xz*_ value but larger ω_*xy*_ value. By comparison, **Pc**:PTP has an estimated κ_rates_ value of 0.3, enough to facilitate the operation of a quasi-CW device pumped invasively using a luminescence concentrator.^7^

### 2.6 Q-Boosted ZF Maser

To finally determine if these materials were suitable as maser gain media, a strontium titanate (STO) resonator suitable for operation between 1700 and 1800 MHz was fabricated (see the SI for details). Compared to alternative materials, STO is known to yield high native quality factors (*Q*_F_, ∼2000 at 1765 MHz) while maintaining a relatively small resonator footprint due to a high relative permittivity resulting in a compressed magnetic mode volume and an enhanced Purcell effect.^6^ By inserting the resonator into a copper cavity with an adjustable height, the resonant frequency could be adjusted to accommodate the transition frequencies for all three azatetracene derivatives.

Initially, native *Q*_L_ pulsed maser tests were conducted using pump energies up to 40 mJ/pulse with 520 or 530 nm light, however, no maser signals could be detected. We attribute this to the broad (∼40 MHz) transition linewidths revealed by ZF trEPR spectroscopy. For masers, the consequence is a lower microwave power output per unit frequency, and, therefore, compared to **Pc**:PTP, the maser signal would be significantly reduced assuming identical operating conditions. The bandwidth of our STO resonator between 1755 and 1765 MHz was measured to be ∼800 kHz from which it can be inferred that much of the microwave output from our materials would not fall within the resonance bandwidth. Thus, if one could dope these materials into a crystal matrix and consequently narrow the emission linewidth, masing could be significantly easier.

Therefore, to effectively determine the cooperativity enhancement that would be required to reach the maser threshold, Q-boosting experiments were performed (see the SI for details). Here, briefly, the output power of the resonator was outcoupled into a feedback loop that amplified the microwave circuit power. This compensated for radiative losses from the resonator cavity and resulted in an artificially increased *Q*_L_.^57^ By adjusting the attenuation of the circuit, the *Q*_L_ of the maser cavity could be willingly increased until circuit self-oscillation, which generally occurred when *Q*_L_ ≥ 220,000 between 1755 and 1765 MHz. For less intense maser bursts, the *Q*_L_ required adjustment closer to the self-oscillation threshold.

**Figure 5:**
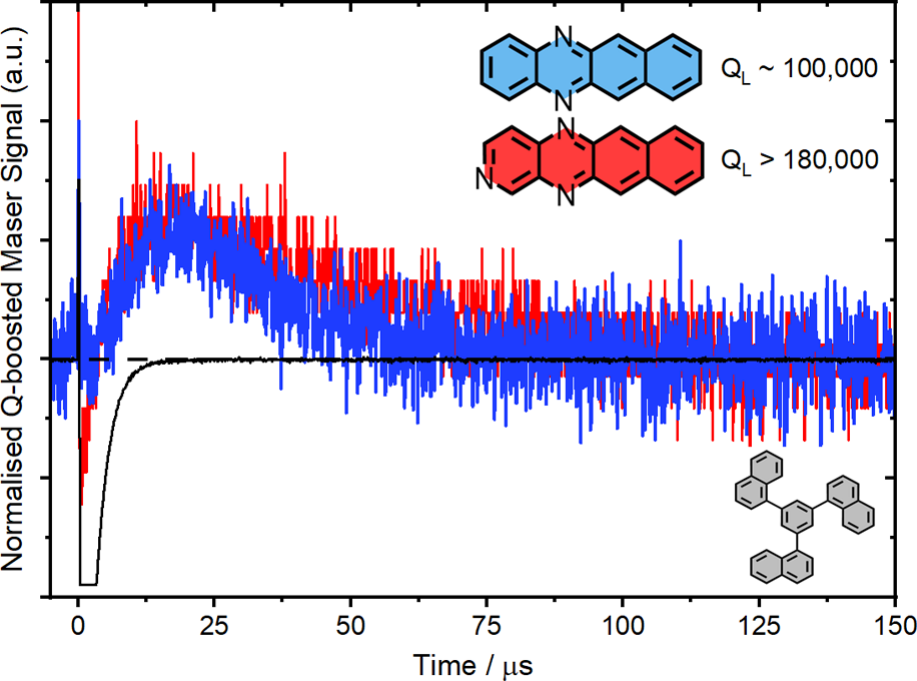
Q-boosted maser signal for **DAT**:1-TNB (blue) at 1765 MHz and **TrAT2**:1-TNB (red) at 1755 MHz. Samples were made at a 0.1% concentration. Laser trigger is represented by the black line. Quality factors were measured using a vector network analyzer. To check whether the signals were sample-dependent, the experiments were repeated with a permanent magnet positioned close to the cavity resulting in the disappearance of the signal.

In this configuration, single-shot maser oscillations were observed for **DAT**:1-TNB and **TrAT2**:1-TNB samples, but not for **TrAT1**:1-TNB (Figure 5). **DAT**:1-TNB required a *Q*_L_ of ∼100,000 to reach the threshold, whereas **TrAT2**:1-TNB required *Q*_L_ > 180,000—with the circuit on the verge of self-oscillation. The maser pulse of **TrAT2**:1-TNB lasted approximately ∼75 μs compared with ∼50 μs for **DAT**:1-TNB. Since the polarization lifetimes measured at ZF were approximately the same, we attributed the difference in burst duration to the different effective *Q*_L_. Overall, while **DAT**:1-TNB and **TrAT2**:1-TNB were found to yield Q-boosted maser signals, an extremely high *Q*_L_ was required, making any prospect of masing with native-Q resonators unrealistic in the current 1-TNB host.

## 3 Discussion

Despite their discovery over a decade ago, organic maser gain media have remained largely undeveloped, hindering the advancement of low pump threshold and high-gain maser technology. Since the operation of these devices relies on the generation of strong electron spin polarization, we sought to chemically modulate and evaluate the maser potential of three related azatetracene compounds, namely, **DAT**, **TrAT1**, and **TrAT2,** doped into a universal organic glass host, 1-TNB. Indeed, the strongest candidates resulting from this work were **DAT**:1-TNB and **TrAT2**:1-TNB, which each demonstrated unique dynamical spin behavior conducive toward masing.

Intelligent chemical approaches to the design of maser host materials have yet to be significantly explored for masers. Hosts such as *p*-terphenyl offer a structurally well-defined host matrix where dopants become substituted in lattice sites. Compared to an amorphous host, this has the benefit of reducing the inhomogeneous broadening of electron spin transitions.^58^ However, such hosts are extremely selective toward dopants with polarity and size-dependent exclusion mechanisms often leading to the elimination of dopants. As a result, the development of crystalline maser materials remains highly challenging. The host material 1-TNB, although amorphous, permits the evaluation of any soluble organic candidate gain material, including various spin-polarized molecules from the existing literature. Hence, it should serve to make the field more accessible since most maser-related properties can be evaluated using commercial instruments. In practice, if a promising maser material is revealed in 1-TNB, additional resources could thereafter be directed toward finding a crystalline host.

Since the pump frequency (*f*_opt_, eq 2) for maser devices is inversely proportional to their cooperativity, hosts that induce an absorption red-shift without adversely affecting the generation of triplets are expected to dulcify the operating conditions. Stronger red shifts can be achieved through strong guest–host charge-transfer interactions, as has been seen in the context of masers with phenazine:TCNB, however further work is needed to understand the impact of charge-transfer interactions and the mobile nature of triplet states for maser applications.^57^ Compared to **Pc**:PTP, which is pumped with 590 nm, illumination with 530 nm light represents an expected ∼10% loss of cooperativity. Nevertheless, this also served to place the longest wavelength absorption bands firmly within the range of commercial 532 nm light sources and facilitates the use of powerful OPO laser emission bands for future maser experiments. In principle, the host can be used to realize more efficient and diverse pumping schemes. Alternative crystalline hosts for pentacene, such as benzoic acid,^59−62^ naphthalene,^63,64^ anthracene,^65,66^ or tetracene,^67^ are known to modulate the triplet dynamics but have not been demonstrated to exhibit any enhanced maser-related properties compared to *p*-terphenyl. However, films and single crystals of pentacene-doped picene (1% mol/mol) exhibit delayed modes of optical transfer from the picene host to the pentacene dopant.^68,69^ Pentacene-doped *trans*-1,4-distyrylbenzene (*trans*-DSB) shows similar guest–host optical transfer activity, but neither has thus far been directly operated in maser devices.^70^ Whether the delayed optical emissions from these materials is matched by extended spin polarization lifetimes is yet to be determined.

Simultaneously to host development, it is essential that new spin-active compounds are found. Here, the fastest relaxation of S_1_ states occurred in the order of **TrAT1**:1-TNB > **DAT**:1-TNB > **TrAT2**:1-TNB. Since within structurally similar compounds the relative abruptness of S_1_ relaxation is associated with the rate of ISC from S_1_ → T_*n*_ states,^23,30,32,71^ it can be assumed that this order also represents the relative abundance of triplet states following photoexcitation. This is supported by the detection of triplet absorption bands for **TrAT1** and **DAT**, but not **TrAT2**, and the corresponding reduced Φ_F_ for **TrAT1**:1-TNB and **DAT**:1-TNB. Interestingly, compared to our materials, tetracene appears to exhibit a stronger preference toward triplet formation.^30,32,33^ However, 5-azatetracene exhibits 63% Φ_F_ when measured in dichloromethane,^72^ suggesting that the structures of di- or tri-azatetracenes derivatives are likely still better at forming triplets than this monosubstituted compound. While more efficient ISC has been identified to result from the introduction of N-heteroatoms, this has usually been attributed to an acceleration of singlet fission^73−75^ and solvent-induced/intermolecular vibronic or spin-orbit coupling.^51,76−79^ Since our dopant-based materials consist of diluted spins, bimolecular processes such as singlet fission or triplet–triplet annihilation can be ruled out. Hence, in our materials, the efficiency of ISC is dominated by variations in Δ*E*_ST_. It is worth mentioning that a precise evaluation of singlet–triplet energies is a particularly challenging subject, especially in the solid state where modeling of guest–host interactions requires a bespoke model.^11,12^ Since 1-TNB is a glass-forming host, this approach is not easily feasible. Therefore, while our DFT calculations managed to reproduce the experimental trends, the absolute values are likely inaccurate. An important challenge for the design of maser compounds is a vigorous and accessible methodology for preliminary computational evaluations.

Investigations involving systematic variations within structurally similar compounds remain a cost-effective approach. Here, for example, similar ZFS parameters permitted maser testing with the same high-Q dielectric resonator. This investigation demonstrated that a high triplet yield must be balanced with the degree of spin polarization. Despite the fastest apparent rate of ISC, **TrAT1**:1-TNB exhibited the smallest T_*x*_:T_*z*_ population difference out of the three materials and as a result could not detectably mase. Whilst maser cooperativity is effectively proportional to the Φ_T_,^9^ there is limited room for improvement in this parameter compared to **Pc**:PTP, for example, where Φ_T_ = 62.5%.^80^ More substantial cooperativity enhancements can be realized by enhancing spin polarization. Organic materials are especially suited for such an approach, with the T_*z*_ population approaching virtually nil for some reported systems.^81−83^ However, since the cooperativity dependence on spin polarization is captured within *T*_1_^eff^, a more comprehensive spin dynamics investigation is required at ZF.^4^ An important challenge is the alleviation of the polarization “bottleneck” associated with **Pc**:PTP that prevents CW masing.^4^ This must also be matched by a relatively narrow microwave emission band, which as this study has demonstrated can be fatal even for pulsed masers. It is notable that despite a relatively poor steady-state spin polarization, dilute spin concentration (≤10^–4^%), and hyperfine splitting, negatively charged nitrogen-vacancy (NV^–^) diamond can mase due to its narrow transition linewidth (1.7 MHz).^56,84^

## 4 Conclusions

Organic spin systems were the first to be used in room-temperature solid-state maser devices. Uniquely, these systems are tunable by modification of their chemical structure and can thus potentially lead to the synthesis of low pump threshold, high-gain maser devices. Using a universal organic host, our findings systemically demonstrate that careful modification of an azatetracene family of compounds leads to measurable consequences for maser applications. Critically, the cooperativity of candidate materials is a function of spin transition linewidth, triplet yield, triplet sublevel-selective decay rate, spin polarization strength, and lifetime. Therefore, the majority of maser-related properties can be evaluated without the use of bespoke built ZF spectrometers and high-Q resonators. Future development of organic systems should seek to not only synthetically develop materials with enhanced spin dynamics and higher spin concentrations, but also incorporate innovative pump schemes. This highly cross-disciplinary topic is therefore open to fruitful contributions from various chemical and engineering teams interested in the development of novel photonic quantum technologies.

## 5 Experimental Section/Methods

Sample preparations: 1-TNB (>98%) was purchased from TCI chemicals. Trace colored impurities were removed by column chromatography using an ethyl acetate: hexane (1:100) eluent system.

**DAT**, **TrAT1,** and **TrAT2** were synthesized according to ref (21). Briefly, 1,2-dihydroxylnaphthalene was ground with a molar equivalent of the corresponding 1,2-diaminopyridine (for **DAT**) or 1,2-diaminopyrimidine (for **TrAT1** & **TrAT2**) in a pestle and mortar. The mixture was then heated in a round-bottomed flask at 160 °C under argon for 1 h, before being cooled and stirred with acetone to produce a slurry. The suspension was then filtered to yield the crude 5,12-dihydrobenzo- or pyrido-quinoxaline precursors as yellow/green solids. The precursors were co-dissolved with a molar equivalent of chloranil in toluene without further purification and refluxed for 1 h under air before being filtered hot. The filtrate was quickly diluted with excess toluene to prevent the crystallization of co-crystals with 1,2,4,5-tetrachloro-3,6-dihydroxyquinone (TCHQ). TCHQ was removed by extraction with an excess of 1 M NaOH solution until no further precipitation occurred in the extraction funnel. The toluene layer was then dried over Na_2_SO_4_ and removed under reduced pressure. Further purification was then performed by sublimation at 110 °C in a nitrogen atmosphere at a typical pressure of 10^–3^ mbar and flow rate of 30 mL/min.

UV/Vis spectroscopy was performed using a sealed 1 cm quartz cuvette filled with samples diluted to 10^–5^ M. Data were measured using a Agilent Cary 5000 UV/Vis/NIR spectrophotometer. Fluorescence emission and excitation spectroscopy were performed using a Horiba Jobin-Yvon Spex Fluorolog-3 fluorimeter, with emission and excitation slit widths set to 2 nm.

For TCSPC and fsTAS experiments, **DAT**, **TrAT1**, and **TrAT2**-doped 1-TNB samples were measured inside rectangular 60 × 4 × 0.4 mm glass tubes (VitroTubes, CMScientific). To load the samples, doped 1-TNB powders were loaded into glass vials made by sealing the end of a glass Pasteur pipette. The capillary tube was pushed into the powder and the whole vial was placed under an inert argon atmosphere inside a larger glass tube. The powder was then carefully melted causing the materials to passively creep into the tube by capillary action. The system was then allowed to cool resulting in the doped 1-TNB samples forming as transparent glasses inside the capillary tubes. Over hours or days, the samples lose their transparency which can be easily restored by remelting the material, taking care not to overheat and force the material to leak out of the tube. When kept in the dark, these samples were found to retain a strong characteristic absorption spectrum over several months. Solution phase measurements were performed in 1 mm quartz cuvettes.

Time-resolved (tr) X-band EPR spectroscopy was performed using a Bruker ELEXSYS-II E500 spectrometer (X-band) at room temperature. Powder samples were decanted into a 4 mm O.D. Wilmad quartz (CFQ) EPR tube and reformed by melting/cooling under an inert argon atmosphere to reduce light scattering. All data were processed using the EasySpin 6.0.0 package for Matlab 2022b (see the SI for details). The least squares fitting was performed using the esfit function and performed with data “as is” using the Nelder/Mead simplex algorithm. The fitting of all three materials was initialized by assuming a Lorentzian lineshape, spin polarization, and ZFS parameters for **DAT** reported by Kouno et al., 2019^18^ and a *g*-value of 2.0023.

ZF trEPR experiments were performed using a home-built spectrometer. The configuration was similar to that in our previously published paper.^4^ For operation at 1.7 GHz, frequency-dependent components including circulators, bandpass filters, and attenuators were changed to fit the resonate frequency. An OPO (Litron Aurora II Integra) pumped by its own internal Q-switched Nd/YAG laser was used to excite the sample. For **DAT**:1-TNB, the output wavelength of the OPO was set to 510 nm with a repetition frequency of 10 Hz and a duration of 5.5 ns. For **TrAT1**:1-TNB and **TrAT2**:1-TNB, the output was adjusted to 530 nm. After being attenuated by three optical filters, the incident illumination energy on the sample was approximately 1 mJ/pulse. The sample was placed in an IC resonator, with an area and the number of turns adjusted for each transition frequency (e.g., T_*x*_ → T_*y*_; T_*x*_ → T_*z*_; T_*y*_ → T_*z*_). The corresponding signals were captured using I-Q homodyne detection recorded in an oscilloscope (Rigol DS1104 Z-plus) with a total of 512 averages.

The photoluminescence spectra for measurements of the fluorescence quantum yield (Φ_F_) were recorded using a home-built system by integrating a charge-coupled device camera (Andor DU420A-BEX2-DD, Oxford Instrument), a spectrograph (Andor KY193, Oxford Instrument), and an integrating sphere (AvaSphere-50-REFL, Avantes). Collimated laser diode modules (CPS532 and CPS405, THORLABS) were used as excitation sources for 532 (50 mW cm^–2^) and 405 nm (50 mW cm^–2^). The system was calibrated by shining two light sources into the integrating sphere with known spectra. A mercury light calibration source (AvaLight-CAL-MINI, AVANTES) was used for wavelength correction, and a halogen light source (AvaLight-HALCAL-ISP50-MINI, AVANTES) was used for the absolute photon flux calibration. The spectral photon density of the corrected spectrum was then divided by the photon energy followed by a numerical integration to obtain the absolute photon counts for the total emission. The Φ_F_ was determined by dividing the photoluminescence photon numbers by the absorbed photon numbers.

## Data Availability

The data that support the findings of this study are available in the supplementary material or upon reasonable request.

## Supporting Information Available

Further characterization by UV/Vis and fluorescence spectroscopy, transient fluorescence, quantum chemical calculations, and ZF EPR spectroscopy, fitting of fsTAS and EPR data, and details of the Q-boost maser experiments (PDF)

## References

[ref1] Mollier J. C.; Hardin J.; Uebersfeld J. Theoretical and Experimental Sensitivities of ESR Spectrometers Using Maser Techniques. Rev. Sci. Instrum. 1973, 44, 1763–1771. 10.1063/1.1686050.

[ref2] Blank A.; Levanon H. Toward Maser Action at Room Temperature by Triplet-Radical Interaction and Its Application to Microwave Technology. RIKEN Rev. 2002, 44, 128.

[ref3] Arroo D. M.; Alford N. M.; Breeze J. D. Perspective on Room-Temperature Solid-State Masers. Appl. Phys. Lett. 2021, 119, 140502 10.1063/5.0061330.

[ref4] Wu H.; Ng W.; Mirkhanov S.; Amirzhan A.; Nitnara S.; Oxborrow M. Unraveling the Room-Temperature Spin Dynamics of Photoexcited Pentacene in Its Lowest Triplet State at Zero Field. J. Phys. Chem. C 2019, 123, 24275–24279. 10.1021/acs.jpcc.9b08439.

[ref5] Oxborrow M.; Breeze J. D.; Alford N. M. Room-Temperature Solid-State Maser. Nature 2012, 488, 353–356. 10.1038/nature11339. 22895341

[ref6] Breeze J.; Tan K.-J.; Richards B.; Sathian J.; Oxborrow M.; Alford N. M. Enhanced Magnetic Purcell Effect in Room-Temperature Masers. Nat. Commun. 2015, 6, 6215 10.1038/ncomms7215. 25698634 PMC4346616

[ref7] Wu H.; Xie X.; Ng W.; Mehanna S.; Li Y.; Attwood M.; Oxborrow M. Room-Temperature Quasi-Continuous-Wave Pentacene Maser Pumped by an Invasive Ce:YAG Luminescent Concentrator. Phys. Rev. Appl. 2020, 14, 064017 10.1103/PhysRevApplied.14.064017.

[ref8] Sathian J.; Breeze J. D.; Richards B.; Alford N. M.; Oxborrow M. Solid-State Source of Intense Yellow Light Based on a Ce:YAG Luminescent Concentrator. Opt. Express 2017, 25, 13714 10.1364/OE.25.013714. 28788914

[ref9] Oxborrow. US Patent Maser Assembly No. 9608396, 2015.

[ref10] Bogatko S.; Haynes P. D.; Sathian J.; Wade J.; Kim J. S.; Tan K. J.; Breeze J.; Salvadori E.; Horsfield A.; Oxborrow M. Molecular Design of a Room-Temperature Maser. J. Phys. Chem. C 2016, 120, 8251–8260. 10.1021/acs.jpcc.6b00150.

[ref11] Charlton R. J.; Fogarty R. M.; Bogatko S.; Zuehlsdorff T. J.; Hine N. D. M.; Heeney M.; Horsfield A. P.; Haynes P. D. Implicit and Explicit Host Effects on Excitons in Pentacene Derivatives. J. Chem. Phys. 2018, 148, 104108 10.1063/1.5017285. 29544310

[ref12] Bertoni A. I.; Fogarty R. M.; Sánchez C. G.; Horsfield A. P. QM/MM Optimization with Quantum Coupling: Host–Guest Interactions in a Pentacene-Doped p -Terphenyl Crystal. J. Chem. Phys. 2022, 156, 044110 10.1063/5.0079788. 35105093

[ref13] Ng W.; Xu X.; Attwood M.; Wu H.; Meng Z.; Chen X.; Oxborrow M. Move Aside Pentacene: Diazapentacene Doped Para-Terphenyl, a Zero-Field Room-Temperature Maser with Strong Coupling for Cavity Quantum Electrodynamics. Adv. Mater. 2023, e2300441 10.1002/adma.202300441. 36919948

[ref14] Wu H.; Mirkhanov S.; Ng W.; Chen K.-C.; Xiong Y.; Oxborrow M. Invasive Optical Pumping for Room-Temperature Masers, Time-Resolved EPR, Triplet-DNP, and Quantum Engines Exploiting Strong Coupling. Opt. Express 2020, 28, 29691 10.1364/OE.401294. 33114862

[ref15] Bonvallet P. A.; Breitkreuz C. J.; Kim Y. S.; Todd E. M.; Traynor K.; Fry C. G.; Ediger M. D.; McMahon R. J. Organic Glass-Forming Materials: 1,3,5-Tris(Naphthyl)Benzene Derivatives. J. Org. Chem. 2007, 72, 10051–10057. 10.1021/jo701921m. 18020366

[ref16] Dawson K.; Kopff L. A.; Zhu L.; McMahon R. J.; Yu L.; Richert R.; Ediger M. D. Molecular Packing in Highly Stable Glasses of Vapor-Deposited Tris-Naphthylbenzene Isomers. J. Chem. Phys. 2012, 136, 094505 10.1063/1.3686801. 22401450

[ref17] Schröder M.; Rauber D.; Matt C.; Kay C. W. M. Pentacene in 1,3,5-Tri(1-Naphtyl)Benzene: A Novel Standard for Transient EPR Spectroscopy at Room Temperature. Appl. Magn. Reson. 2022, 53, 1043–1052. 10.1007/s00723-021-01420-4.

[ref18] Kouno H.; Kawashima Y.; Tateishi K.; Uesaka T.; Kimizuka N.; Yanai N. Nonpentacene Polarizing Agents with Improved Air Stability for Triplet Dynamic Nuclear Polarization at Room Temperature. J. Phys. Chem. Lett. 2019, 10, 2208–2213. 10.1021/acs.jpclett.9b00480. 30933529

[ref19] Nishimura K.; Kouno H.; Kawashima Y.; Orihashi K.; Fujiwara S.; Tateishi K.; Uesaka T.; Kimizuka N.; Yanai N. Materials Chemistry of Triplet Dynamic Nuclear Polarization. Chem. Commun. 2020, 56, 7217–7232. 10.1039/d0cc02258f. 32495753

[ref20] Fujiwara S.; Matsumoto N.; Nishimura K.; Kimizuka N.; Tateishi K.; Uesaka T.; Yanai N. Triplet Dynamic Nuclear Polarization of Guest Molecules through Induced Fit in a Flexible Metal–Organic Framework**. Angew. Chem., Int. Ed. 2022, 61, 25–28. 10.1002/anie.202115792. 34935275

[ref21] Attwood M.; Kim D. K.; Hadden J. H. L.; Maho A.; Ng W.; Wu H.; Akutsu H.; White A. J. P.; Heutz S.; Oxborrow M. Asymmetric N-Heteroacene Tetracene Analogues as Potential n-Type Semiconductors. J. Mater. Chem. C 2021, 9, 17073–17083. 10.1039/D1TC03933D.

[ref22] Bossanyi D. G.; Sasaki Y.; Wang S.; Chekulaev D.; Kimizuka N.; Yanai N.; Clark J. Spin Statistics for Triplet–Triplet Annihilation Upconversion: Exchange Coupling, Intermolecular Orientation, and Reverse Intersystem Crossing. JACS Au 2021, 1, 2188–2201. 10.1021/jacsau.1c00322. 34977890 PMC8715495

[ref23] Chen J.; Chen Y.; Wu Y.; Wang X.; Yu Z.; Xiao L.; Liu Y.; Tian H.; Yao J.; Fu H. Modulated Emission from Dark Triplet Excitons in Aza-Acene Compounds: Fluorescence versus Phosphorescence. New J. Chem. 2017, 41, 1864–1871. 10.1039/c6nj02747d.

[ref24] Zhang Y. D.; Wu Y.; Xu Y.; Wang Q.; Liu K.; Chen J. W.; Cao J. J.; Zhang C.; Fu H.; Zhang H. L. Excessive Exoergicity Reduces Singlet Exciton Fission Efficiency of Heteroacenes in Solutions. J. Am. Chem. Soc. 2016, 138, 6739–6745. 10.1021/jacs.6b03829. 27167770

[ref25] Avalos C. E.; Richert S.; Socie E.; Karthikeyan G.; Casano G.; Stevanato G.; Kubicki D. J.; Moser J. E.; Timmel C. R.; Lelli M.; Rossini A. J.; Ouari O.; Emsley L. Enhanced Intersystem Crossing and Transient Electron Spin Polarization in a Photoexcited Pentacene-Trityl Radical. J. Phys. Chem. A 2020, 124, 6068–6075. 10.1021/acs.jpca.0c03498. 32585095

[ref26] Chergui M. Empirical Rules of Molecular Photophysics in the Light of Ultrafast Spectroscopy. Pure Appl. Chem. 2015, 87, 525–536. 10.1515/pac-2014-0939.

[ref27] Demchenko A. P.; Tomin V. I.; Chou P.-T. Breaking the Kasha Rule for More Efficient Photochemistry. Chem. Rev. 2017, 117, 13353–13381. 10.1021/acs.chemrev.7b00110. 28991479

[ref28] Nijegorodov N.; Ramachandran V.; Winkoun D. P. The Dependence of the Absorption and Fluorescence Parameters, the Intersystem Crossing and Internal Conversion Rate Constants on the Number of Rings in Polyacene Molecules. Spectrochim. Acta, Part A 1997, 53, 1813–1824. 10.1016/S1386-1425(97)00071-1.

[ref29] Reindl S.; Penzkofer A. Higher Excited-State Triplet-Singlet Intersystem Crossing of Some Organic Dyes. Chem. Phys. 1996, 211, 431–439. 10.1016/0301-0104(96)00191-7.

[ref30] Burdett J. J.; Müller A. M.; Gosztola D.; Bardeen C. J. Excited State Dynamics in Solid and Monomeric Tetracene: The Roles of Superradiance and Exciton Fission. J. Chem. Phys. 2010, 133, 144506 10.1063/1.3495764. 20950016

[ref31] Lenci F.; Checcucci G.; Sgarbossa A.; Martin M. M.; Plaza P.; Angelini N. Fluorescent Biomolecules. *Encycl*. Condens. Matter Phys. 1999, 2005, 222–235. 10.1016/B0-12-369401-9/01122-0.

[ref32] Burgdorff C.; Kircher T.; Löhmannsröben H. G. Photophysical Properties of Tetracene Derivatives in Solution. Spectrochim. Acta, Part A 1988, 44, 1137–1141. 10.1016/0584-8539(88)80084-9.

[ref33] Kearvell A.; Wilkinson F. Internal Conversion from the Lowest Excited Singlet States of Aromatic Hydrocarbons. Chem. Phys. Lett. 1971, 11, 472–473. 10.1016/0009-2614(71)80387-1.

[ref34] Takeda K.; Takegoshi K.; Terao T. Zero-Field Electron Spin Resonance and Theoretical Studies of Light Penetration into Single Crystal and Polycrystalline Material Doped with Molecules Photoexcitable to the Triplet State via Intersystem Crossing. J. Chem. Phys. 2002, 117, 4940–4946. 10.1063/1.1499124.

[ref35] Li H.; Duan L.; Zhang D.; Dong G.; Wang L.; Qiu Y. Preparation and Spectral Characteristics of Anthracene/Tetracene Mixed Crystals. Sci. China Ser. B Chem. 2009, 52, 181–187. 10.1007/s11426-007-0116-7.

[ref36] De Souza T. G. B.; Vivas M. G.; Mendonça C. R.; Plunkett S.; Filatov M. A.; Senge M. O.; De Boni L. Studying the Intersystem Crossing Rate and Triplet Quantum Yield of Meso-Substituted Porphyrins by Means of Pulse Train Fluorescence Technique. J. Porphyr. Phthalocyanines 2016, 20, 282–291. 10.1142/S1088424616500048.

[ref37] Rajagopal S. K.; Mallia A. R.; Hariharan M. Enhanced Intersystem Crossing in Carbonylpyrenes. Phys. Chem. Chem. Phys. 2017, 19, 28225–28231. 10.1039/c7cp04834c. 29027550

[ref38] Huang T. T.; Li E. Y. Enhanced Spin-Orbit Coupling Driven by State Mixing in Organic Molecules for OLED Applications. Org. Electron. 2016, 39, 311–317. 10.1016/j.orgel.2016.10.026.

[ref39] Ompong D.; Singh J. Study of Intersystem Crossing Mechanism in Organic Materials. Phys. Status Solidi 2016, 13, 89–92. 10.1002/pssc.201510128.

[ref40] Marian C. M. Understanding and Controlling Intersystem Crossing in Molecules. Annu. Rev. Phys. Chem. 2021, 72, 617–640. 10.1146/annurev-physchem-061020-053433. 33607918

[ref41] Shafikov M. Z.; Zaytsev A. V.; Kozhevnikov V. N. Halide-Enhanced Spin-Orbit Coupling and the Phosphorescence Rate in Ir(III) Complexes. Inorg. Chem. 2021, 60, 642–650. 10.1021/acs.inorgchem.0c02469. 33405901

[ref42] Alotibi S.; Hickey B. J.; Teobaldi G.; Ali M.; Barker J.; Poli E.; O’Regan D. D.; Ramasse Q.; Burnell G.; Patchett J.; Ciccarelli C.; Alyami M.; Moorsom T.; Cespedes O. Enhanced Spin-Orbit Coupling in Heavy Metals via Molecular Coupling. ACS Appl. Mater. Interfaces 2021, 13, 5228–5234. 10.1021/acsami.0c19403. 33470108

[ref43] Qiu W.; Cai X.; Chen Z.; Wei X.; Li M.; Gu Q.; Peng X.; Xie W.; Jiao Y.; Gan Y.; Liu W.; Su S. J. A “Flexible” Purely Organic Molecule Exhibiting Strong Spin-Orbital Coupling: Toward Nondoped Room-Temperature Phosphorescence OLEDs. J. Phys. Chem. Lett. 2022, 13, 4971–4980. 10.1021/acs.jpclett.2c01205. 35639995

[ref44] Liu H.; Gao Y.; Cao J.; Li T.; Wen Y.; Ge Y.; Zhang L.; Pan G.; Zhou T.; Yang B. Efficient Room-Temperature Phosphorescence Based on a Pure Organic Sulfur-Containing Heterocycle: Folding-Induced Spin-Orbit Coupling Enhancement. Mater. Chem. Front. 2018, 2, 1853–1858. 10.1039/c8qm00320c.

[ref45] Braga H. C.; Salla C. A. M.; Bechtold I. H.; Bortoluzzi A. J.; Souza B.; Gallardo H. The Effect of Spin-Orbit Coupling on Selenadiazolo- and Thiadiazolo- Fused 1,10-Phenanthrolines. Dyes Pigm. 2015, 117, 149–156. 10.1016/j.dyepig.2015.02.012.

[ref46] Rodriguez-Serrano A.; Rai-Constapel V.; Daza M. C.; Doerr M.; Marian C. M. Internal Heavy Atom Effects in Phenothiazinium Dyes: Enhancement of Intersystem Crossing via Vibronic Spin-Orbit Coupling. Phys. Chem. Chem. Phys. 2015, 17, 11350–11358. 10.1039/c5cp00194c. 25845532

[ref47] Mońka M.; Grzywacz D.; Hoffman E.; Ievtukhov V.; Kozakiewicz K.; Rogowski R.; Kubicki A.; Liberek B.; Bojarski P.; Serdiuk I. E. Decisive Role of Heavy-Atom Orientation for Efficient Enhancement of Spin-Orbit Coupling in Organic Thermally Activated Delayed Fluorescence Emitters. J. Mater. Chem. C 2022, 10, 11719–11729. 10.1039/d2tc01729f.

[ref48] Crespo-Hernández C. E.; Burdzinski G.; Arce R. Environmental Photochemistry of Nitro-PAHs: Direct Observation of Ultrafast Intersystem Crossing in 1-Nitropyrene. J. Phys. Chem. A 2008, 112, 6313–6319. 10.1021/jp803847q. 18572893

[ref49] Zugazagoitia J. S.; Almora-Díaz C. X.; Peon J. Ultrafast Intersystem Crossing in 1-Nitronaphthalene. An Experimental and Computational Study. J. Phys. Chem. A 2008, 112, 358–365. 10.1021/jp074809a. 18166024

[ref50] Baba M. Intersystem Crossing in the 1 Nπ* and 1 Ππ* States. J. Phys. Chem. A 2011, 115, 9514–9519. 10.1021/jp111892y. 21401029

[ref51] El-Sayed M. A. Spin—Orbit Coupling and the Radiationless Processes in Nitrogen Heterocyclics. J. Chem. Phys. 1963, 38, 2834–2838. 10.1063/1.1733610.

[ref52] Stoll S.; Schweiger A. EasySpin, a Comprehensive Software Package for Spectral Simulation and Analysis in EPR. J. Magn. Reson. 2006, 178, 42–55. 10.1016/j.jmr.2005.08.013. 16188474

[ref53] Yang T. C.; Sloop D. J.; Weissman S. I.; Lin T. S. Zero-Field Magnetic Resonance of the Photo-Excited Triplet State of Pentacene at Room Temperature. J. Chem. Phys. 2000, 113, 11194–11201. 10.1063/1.1326069.

[ref54] Bayliss S. L.; Kraffert F.; Wang R.; Zhang C.; Bittl R.; Behrends J. Tuning Spin Dynamics in Crystalline Tetracene. J. Phys. Chem. Lett. 2019, 10, 1908–1913. 10.1021/acs.jpclett.9b00356. 30939019

[ref55] Wu H.; Yang S.; Oxborrow M.; Jiang M.; Zhao Q.; Budker D.; Zhang B.; Du J. Enhanced Quantum Sensing with Room-Temperature Solid-State Masers. Sci. Adv. 2022, 8, eade1613 10.1126/sciadv.ade1613. 36449621 PMC9710876

[ref56] Breeze J. D.; Salvadori E.; Sathian J.; Alford N. M. N.; Kay C. W. M. Continuous-Wave Room-Temperature Diamond Maser. Nature 2018, 555, 493–496. 10.1038/nature25970. 29565362

[ref57] Ng W.; Zhang S.; Wu H.; Nevjestic I.; White A. J. P.; Oxborrow M. Exploring the Triplet Spin Dynamics of the Charge-Transfer Co-Crystal Phenazine/1,2,4,5-Tetracyanobenzene for Potential Use in Organic Maser Gain Media. J. Phys. Chem. C 2021, 125, 14718–14728. 10.1021/acs.jpcc.1c01654.

[ref58] Kveder M.; Rakvin B.; Jokić M.; Reijerse E. Frozen-in Disorder Probed by Electron Spin Relaxation. Solid State Commun. 2013, 167, 23–26. 10.1016/j.ssc.2013.04.034.

[ref59] Miyanishi K.; Segawa T. F.; Takeda K.; Ohki I.; Onoda S.; Ohshima T.; Abe H.; Takashima H.; Takeuchi S.; Shames A. I.; Morita K.; Wang Y.; So F. T.-K.; Terada D.; Igarashi R.; Kagawa A.; Kitagawa M.; Mizuochi N.; Shirakawa M.; Negoro M. Room-Temperature Hyperpolarization of Polycrystalline Samples with Optically Polarized Triplet Electrons: Pentacene or Nitrogen-Vacancy Center in Diamond?. Magn. Reson. 2021, 2, 33–48. 10.5194/mr-2-33-2021. PMC10539752 37904782

[ref60] Kryschi C.; Wagner B.; Gorgas W.; Schmid D. Vibronically Induced Intersystem Crossing in Pentacene in P-Terphenyl and Benzoic Acid Crystals. J. Lumin. 1992, 53, 468–472. 10.1016/0022-2313(92)90200-S.

[ref61] Ong J. L.; Sloop D. J.; Lin T. S. Temperature Dependence Studies of the Paramagnetic Properties of the Photoexcited Triplet State of Pentacene in P-Terphenyl, Benzoic Acid, and Naphthalene Crystals. J. Phys. Chem. 1993, 97, 7833–7838. 10.1021/j100132a008.

[ref62] Ong J. L.; Sloop D. J.; Lin T. S. Peculiar Spin Dynamics of the Photoexcited Triplet State of Pentacene in a Benzoic Acid Crystal: An ESE Study. Appl. Magn. Reson. 1994, 6, 359–371. 10.1007/BF03162629.

[ref63] Eichhorn T. R.; Van Den Brandt B.; Hautle P.; Henstra A.; Wenckebach W. T. Dynamic Nuclear Polarisation via the Integrated Solid Effect II: Experiments on Naphthalene-H8 Doped with Pentacene-D14. Mol. Phys. 2014, 112, 1773–1782. 10.1080/00268976.2013.863405.

[ref64] Van Strien A. J.; Schmidt J. An EPR Study of the Triplet State of Pentacene by Electron Spin-Echo Techniques and Laser Flash Excitation. Chem. Phys. Lett. 1980, 70, 513–517. 10.1016/0009-2614(80)80115-1.

[ref65] Wakayama N. I.; Wakayama N.; Williams D. F. Electroluminescence in Pentacene Doped Anthracene Crystals. Bull. Chem. Soc. Jpn. 1973, 46, 3395–3399. 10.1246/bcsj.46.3395.

[ref66] Wakayama N. I.; Wakayama N.; Williams D. F. Pulsed and Steady State Electroluminescence of Pentacene Doped Anthracene Crystals. Mol. Cryst. Liq. Cryst. 1974, 26, 275–280. 10.1080/15421407408083105.

[ref67] Burgos J.; Pope M.; Swenberg C. E.; Alfano R. R. Heterofission in Pentacene-Doped Tetracene Single Crystals. Phys. Status Solidi 1977, 83, 249–256. 10.1002/pssb.2220830127.

[ref68] Moro F.; Moret M.; Ghirri A.; Granados del Águila A.; Kubozono Y.; Beverina L.; Cassinese A. Room-Temperature Optically Detected Magnetic Resonance of Triplet Excitons in a Pentacene-Doped Picene Single Crystal. J. Mater. Res. 2022, 37, 1269–1279. 10.1557/s43578-022-00536-y.

[ref69] Toccoli T.; Bettotti P.; Cassinese A.; Gottardi S.; Kubozono Y.; Loi M. A.; Manca M.; Verucchi R. Photophysics of Pentacene-Doped Picene Thin Films. J. Phys. Chem. C 2018, 122, 16879–16886. 10.1021/acs.jpcc.8b02978.

[ref70] Wang H.; Li F.; Gao B.; Xie Z.; Liu S.; Wang C.; Hu D.; Shen F.; Xu Y.; Shang H.; Chen Q.; Ma Y.; Sun H. Doped Organic Crystals with High Efficiency, Color-Tunable Emission toward Laser Application. Cryst. Growth Des. 2009, 9, 4945–4950. 10.1021/cg9007125.

[ref71] Schmidt K.; Brovelli S.; Coropceanu V.; Beljonne D.; Cornil J.; Bazzini C.; Caronna T.; Tubino R.; Meinardi F.; Shuai Z.; Brédas J. L. Intersystem Crossing Processes in Nonplanar Aromatic Heterocyclic Molecules. J. Phys. Chem. A 2007, 111, 10490–10499. 10.1021/jp075248q. 17910425

[ref72] Ghosh A.; Budanovic M.; Li T.; Liang C.; Klein M.; Soci C.; Webster R. D.; Gurzadyan G. G.; Grimsdale A. C. Synthesis of 5-Azatetracene and Comparison of Its Optical and Electrochemical Properties with Tetracene. Asian J. Org. Chem. 2021, 10, 2571–2579. 10.1002/ajoc.202100373.

[ref73] Bunz U. H. F. The Larger Linear N-Heteroacenes. Acc. Chem. Res. 2015, 48, 1676–1686. 10.1021/acs.accounts.5b00118. 25970089

[ref74] Herz J.; Buckup T.; Paulus F.; Engelhart J.; Bunz U. H. F.; Motzkus M. Acceleration of Singlet Fission in an Aza-Derivative of TIPS-Pentacene. J. Phys. Chem. Lett. 2014, 5, 2425–2430. 10.1021/jz501102r. 26277810

[ref75] Chen Y.; Shen L.; Li X. Effects of Heteroatoms of Tetracene and Pentacene Derivatives on Their Stability and Singlet Fission. J. Phys. Chem. A 2014, 118, 5700–5708. 10.1021/jp503114b. 25007000

[ref76] Lim E. C.; Yu J. M. H. Vibronic Spin—Orbit Interactions in Heteroaromatic Molecules. I. Polycyclic Monoazines. J. Chem. Phys. 1967, 47, 3270–3275. 10.1063/1.1712389.

[ref77] Lim E. C.; Yu J. M. H. Vibronic Spin–Orbit Interactions in Heteroaromatic Molecules. II. Phosphorescence of Quinoxaline and Other Diazanaphthalenes. J. Chem. Phys. 1968, 49, 3878–3884. 10.1063/1.1670693.

[ref78] Antheunis D. A.; Schmidt J.; van der Waals J. H. Spin-Forbidden Radiationless Processes in Isoelectronic Molecules: Anthracene, Acridine and Phenazine. Mol. Phys. 1974, 27, 1521–1541. 10.1080/00268977400101291.

[ref79] Gastilovich E. A.; Val’kova G. A.; Ni B. V. Intersystem Crossing in Molecules of Aromatic Compounds with Their Heteroatom on the C2 Symmetry Axis, Acridine. J. Mol. Struct. 1993, 301, 155–174. 10.1016/0022-2860(93)80242-N.

[ref80] Patterson F. G.; Lee H. W. H.; Wilson W. L.; Fayer M. D. Intersystem Crossing from Singlet States of Molecular Dimers and Monomers in Mixed Molecular Crystals: Picosecond Stimulated Photon Echo Experiments. Chem. Phys. 1984, 84, 51–60. 10.1016/0301-0104(84)80005-1.

[ref81] van Wynsberghe E.; Turak A. Candidate Materials as Gain Media in Organic, Triplet-Based, Room-Temperature Masers Targeting the ISM Bands. In Optoelectronics - Advanced Device Structures; InTech, 2017; pp 213–231.

[ref82] Tait C. E.; Bedi A.; Gidron O.; Behrends J. Photoexcited Triplet States of Twisted Acenes Investigated by Electron Paramagnetic Resonance. Phys. Chem. Chem. Phys. 2019, 21, 21588–21595. 10.1039/c9cp04135d. 31539003

[ref83] Clarke R. H.; Frank H. A. Triplet State Radiationless Transitions in Polycyclic Hydrocarbons. J. Chem. Phys. 1976, 65, 39–47. 10.1063/1.432781.

[ref84] Sherman A.; Zgadzai O.; Koren B.; Peretz I.; Laster E.; Blank A. Diamond-Based Microwave Quantum Amplifier. Sci. Adv. 2022, 8, 1–10. 10.1126/sciadv.ade6527. PMC9728959 36475787

