# Supplementary material for: N-Heteroacenes as an Organic Gain Medium for Room-Temperature Masers
Source: Chem Mater. 2023 May 23;35(11):4498–509. doi: 10.1021/acs.chemmater.3c00640 (PMC10268955; doi:10.1021/acs.chemmater.3c00640)
Supplement: Supplementary file 1 — cm3c00640_si_001.pdf [file cm3c00640_si_001.pdf]

Supporting Information

N-heteroacenes as an organic gain medium for room temperature masers

Max Attwood,[1] Xiaotian Xu,[1] Michael Newns,[1] Zhu Meng,[2] Rebecca A. Ingle,[3] Hao Wu,[4] Xi Chen,[1,7] Weidong Xu,[2] Wern Ng,[1] Temitope T. Abiola,[5] Vasilios G. Stavros,[6] Mark Oxborrow[1]*

Steady-State Optical Spectroscopy S1
Transient Fluorescence Spectroscopy S2
Femtosecond Transient Absorption Spectroscopy (fsTAS) S4
Quantum Chemical Calculations S7
Additional Zero-field EPR Spectroscopy S15
Q-boosting Maser Experiments & Dielectric Resonator Design S16

Steady-State Optical Spectroscopy

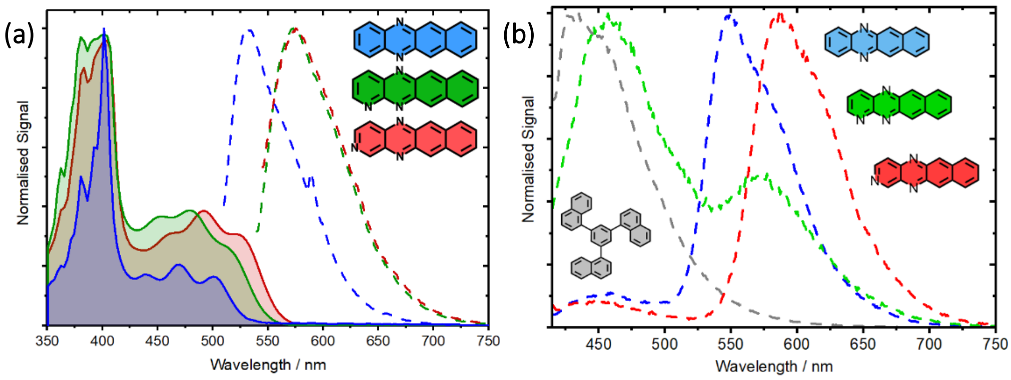

Figure S1. (a) Steady State fluorescence spectroscopy of DAT, TrAT1 and TrAT2 dissolved in THF; (b) Emission spectrum of neat 1-TNB and DAT, TrAT1 and TrAT2-doped 1-TNB samples following excitation at 405 nm. THF concentrations ca. 10^{-4} M; 1-TNB doping concentration 0.1%.

Transient Fluorescence Spectroscopy

TCSPC data was fitting with Origin2021b using the built-in fitting function GaussMod, made up of a single exponential decay function, representing the sample's fluorescence decay, convoluted with a gaussian function, representing the instrument response function (IRF). The best fitting data was determined by a combination of the smallest residual and the highest coefficient of determination (R^2-value). Fitting parameters are given in **Table S1** for the closest fit.

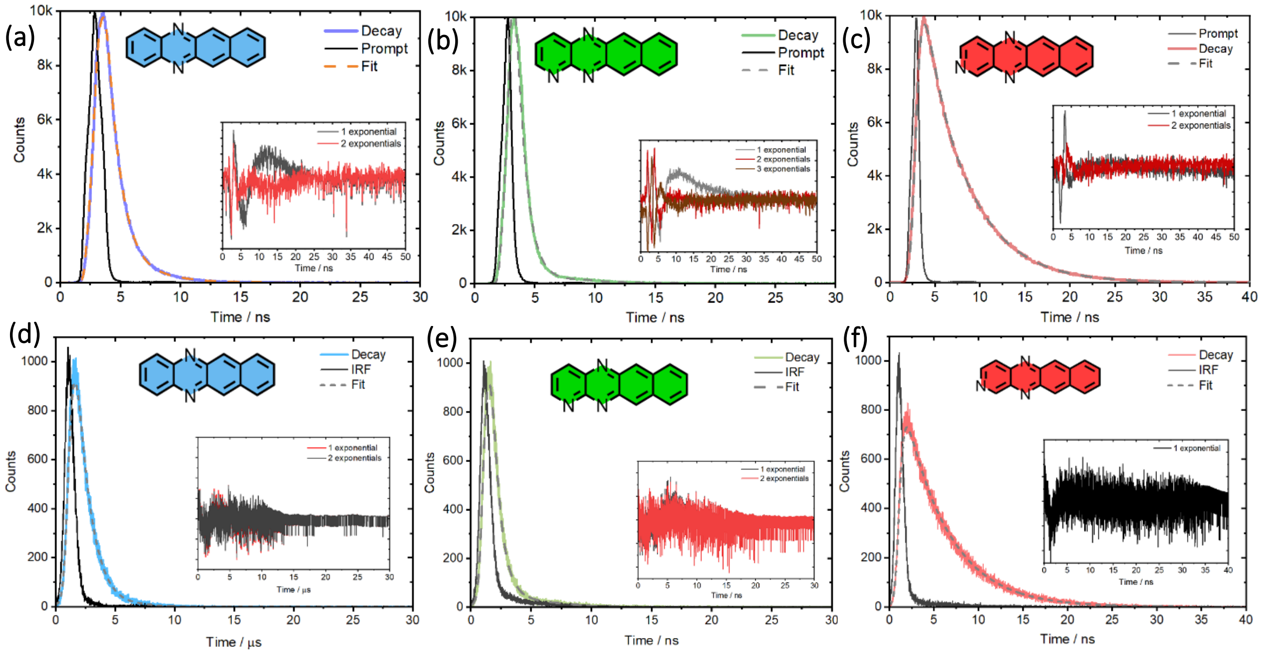

Figure S2. Time-correlated single photon counting (TCSPC) spectroscopy of (a) DAT monitoring emission at 550 nm, (b) TrAT1 and (c) TrAT2 monitoring at 570 nm emission in THF solution. Excitation wavelengths were 530 nm for traces (a-c) and 402 nm for (d-f). Sample concentrations are 10^{-4} M. Residual traces after fitting are displayed in the insets.

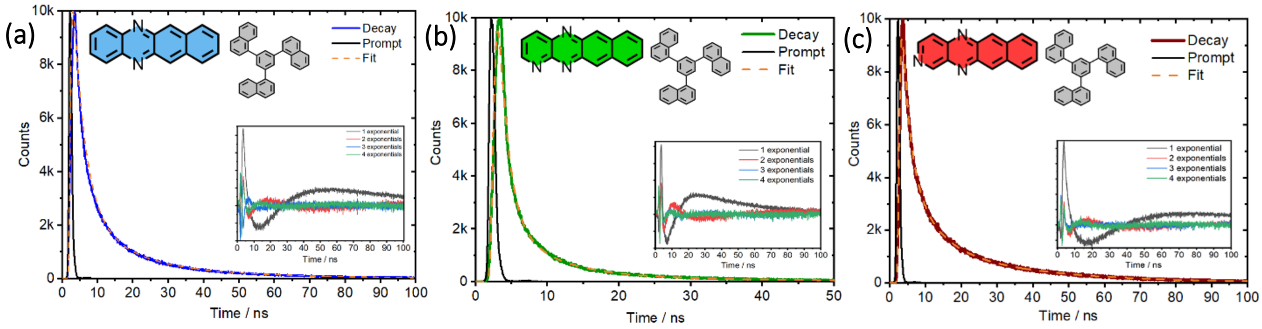

Figure S3. Time-correlated single photon counting (TCSPC) spectroscopy of (a) DAT, (b) TrAT1 and (c) TrAT2 in 1-TNB. Excitation wavelength was 530 nm for all molecules and sample concentrations are 0.1% mol/mol. Residual traces after fitting are displayed in the insets.

Table S1. Fitting parameters for TCSPC data in both THF solution and 1-TNB hosts at various excitation wavelengths. Fittings are provided for the best fit and weighting parameters, $A_{n=1-2}$.

	Host	λ_{ex} / nm	λ_{em} / nm	A_1	τ_1 / ns	A_2	τ_2 / ns	τ_F / ns[a]	R^2
**DAT**	THF	402	550	-	1.09 ± 0.01	-	-	1.09 ± 0.01	0.998
	THF	530	550	0.88	1.13 ± 0.07	0.12	3.16 ± 0.02	1.37 ± 0.05	0.998
	1-TNB	530	560	0.52	4.91 ± 0.04	0.48	24.9 ± 0.14	14.5 ± 0.09	0.993
**TrAT1**	THF	402	570	-	0.597 ± 0.05	-	-	0.597 ± 0.05	0.998
	THF	530	570	-	0.891 ± 0.05	-	-	0.891 ± 0.05	0.993
	1-TNB	530	600	0.62	1.98 ± 0.02	0.38	16.4 ± 0.13	7.46 ± 0.06	0.990
**TrAT2**	THF	402	600	-	4.66 ± 0.02	-	-	4.66 ± 0.02	0.996
	THF	530	600	-	4.77 ± 0.03	-	-	4.77 ± 0.03	0.998
	1-TNB	530	600	0.38	3.86 ± 0.03	0.62	27.4 ± 0.1	18.5 ± 0.07	0.997

Femtosecond Transient Absorption Spectroscopy (fsTAS)

The fsTAS set up employed for the measurements reported herein has been detailed previously.[1] Data was processed and analysed using SurfaceXplorer software (Version 4.3.0). A background subtraction was applied to remove laser scatter, followed by a chirp correction. The data fitted using global analysis (GA) following singular value decomposition (SVD). To find the "best" fit, the number of principal components were increased until the major artefacts of the data were accounted for the residual minimised. Typically, the data for THF and doped 1-TNB samples could be reproduced using two to three components, one of which, usually the 1st, accounted for the laser scatter. Beyond this, additional components accounted for noise, chirp artefacts, residual laser scatter or had weighting < 0.05. Fitting and plotting of the principal component data was performed in Origin 2022b and time profiles were fitted using a mono- or biexponential decay function.

To confirm that these spectral features observed for **DAT**, **TrAT1** and **TrAT2**-doped 1-TNB samples did not originate from the 1-TNB host, its transient absorption spectrum was measured following excitation at 530 nm using a sample from the same chemical batch that was used to make all our samples (**Figure S4**). In this case, the ΔOD spectrum of 1-TNB appears flat without any notable features.

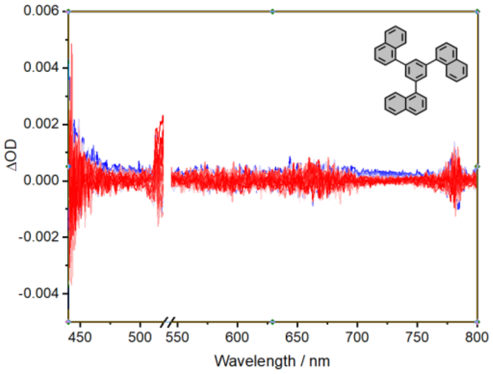

Figure S4: fsTAS data for neat 1-TNB following illumination at 530 nm.

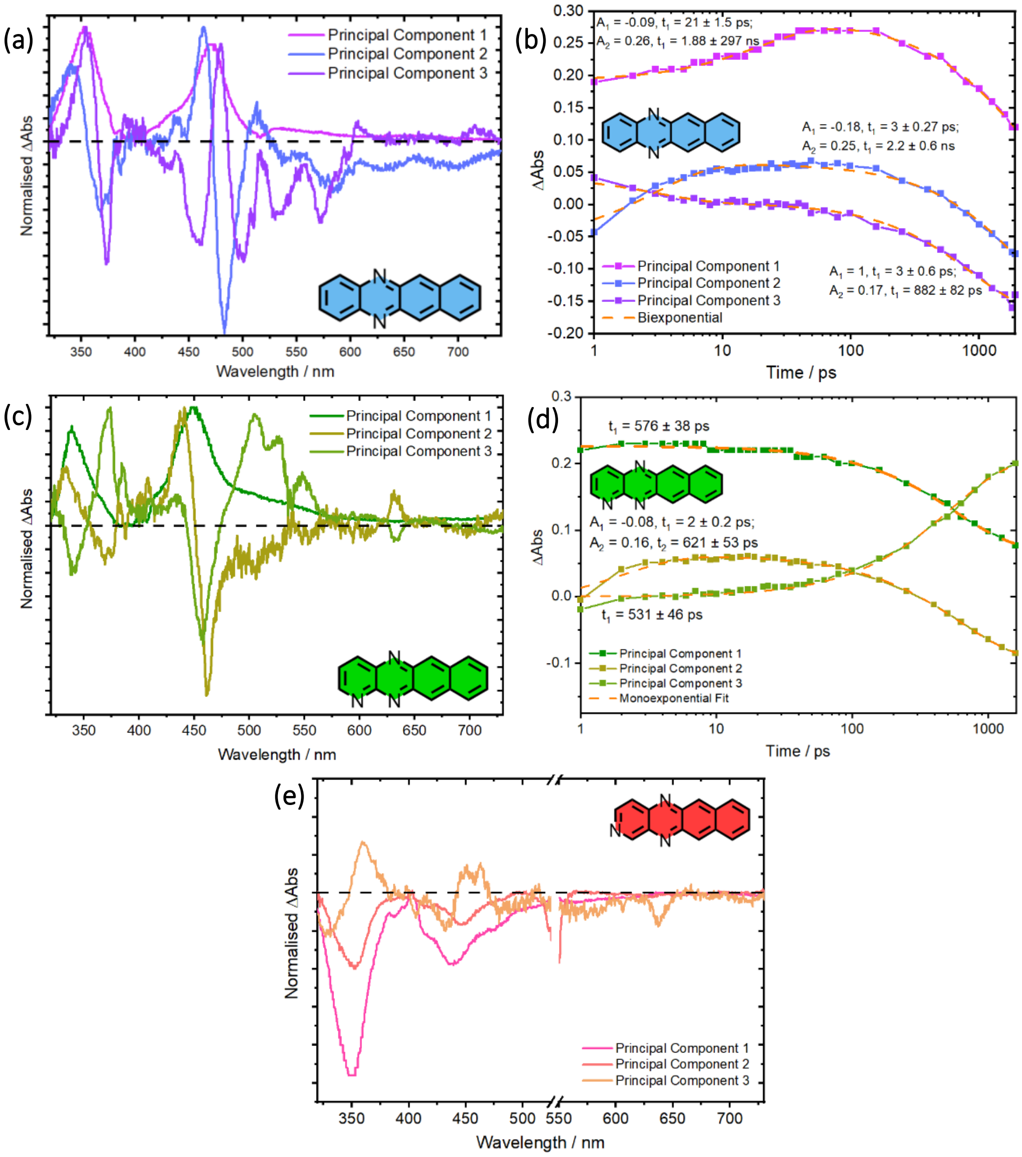

Figure S5: Principal components and fitted kinetics for fsTAS data for (a & b) DAT, (c & d) TrAT1 and (e) TrAT2 in THF following excitation at 530 nm. For **TrAT2**, the progression of excited state decay was not sufficient within the 1.9 ns timespan of the experiment to allow for sensible fitting.

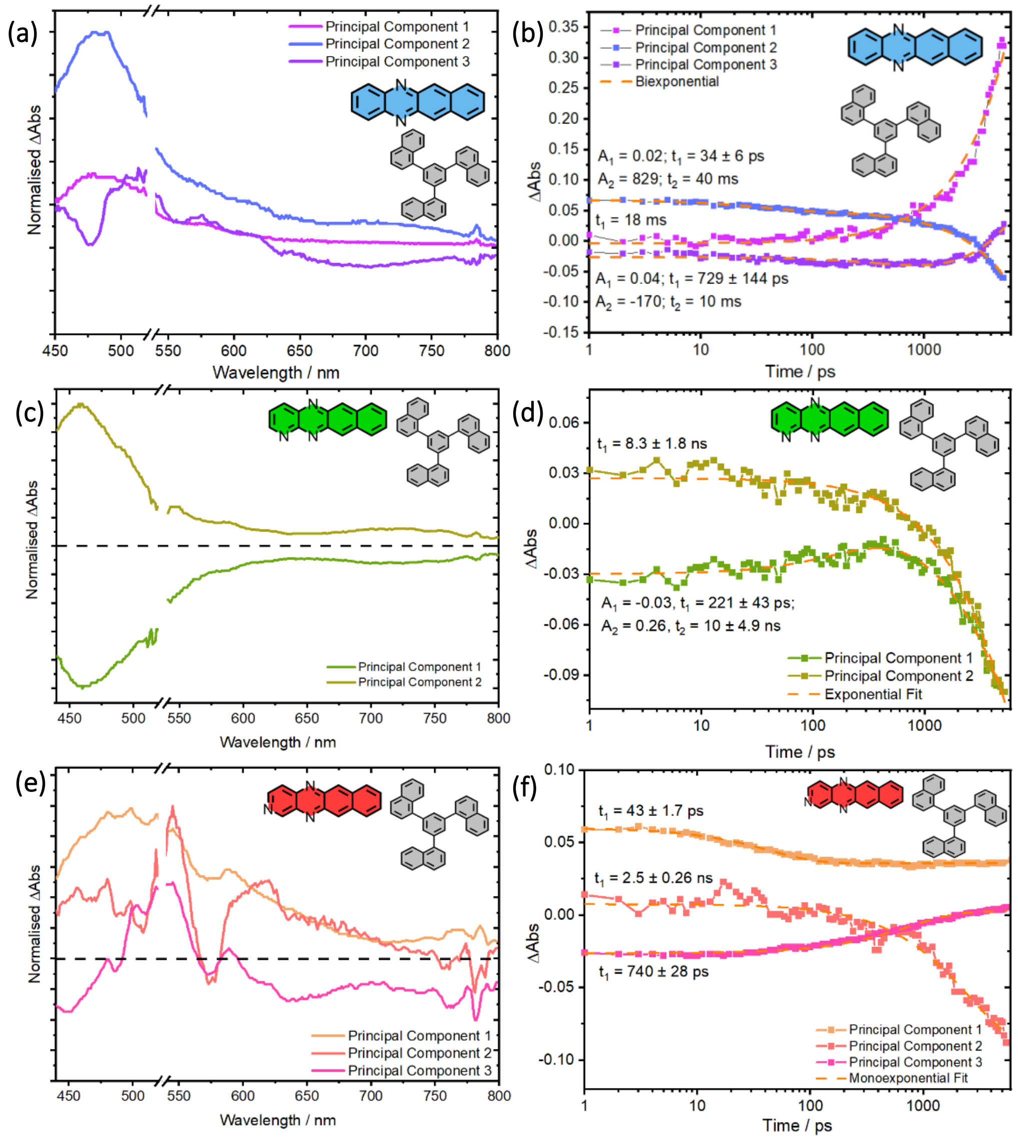

Figure S6. Principal components and fitted kinetics for fsTAS data for (a & b) DAT, (c & d) TrAT1 and (e & f) TrAT2 in 1-TNB following excitation at 530 nm.

Quantum Chemical Calculations

Following an exploration of Density functional theory (DFT) functional and basis set combinations (**Table S2**), quantum calculations were performed in Gaussian09 software[2] at the B3LYP[3] 6-311G++(d,p) level of theory. In each case, the compounds were first optimised in their ground state prior to single point energy calculations that were performed to determine the HOMO-LUMO energy separation. Calculated UV/Vis spectra were obtained as single point excitation energies on the minimum energy structures. Natural transition orbitals (NTOs) were calculated using excited state optimised structures at the same level of theory with an IEFPCM solvent model of benzene.

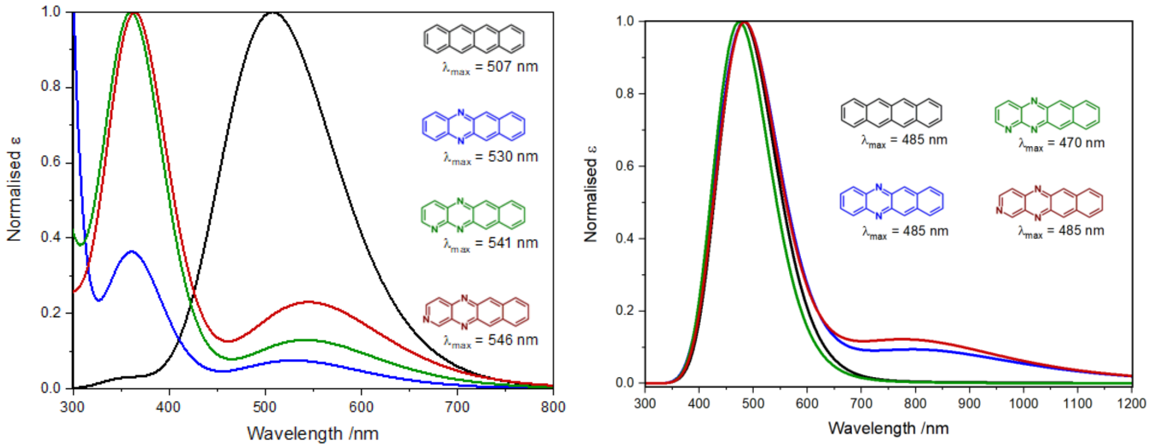

Figure S7. Singlet (left) and triplet (right) UV/Vis spectrum calculated by DFT at the B3LYP 6-311G++(d,p) level.

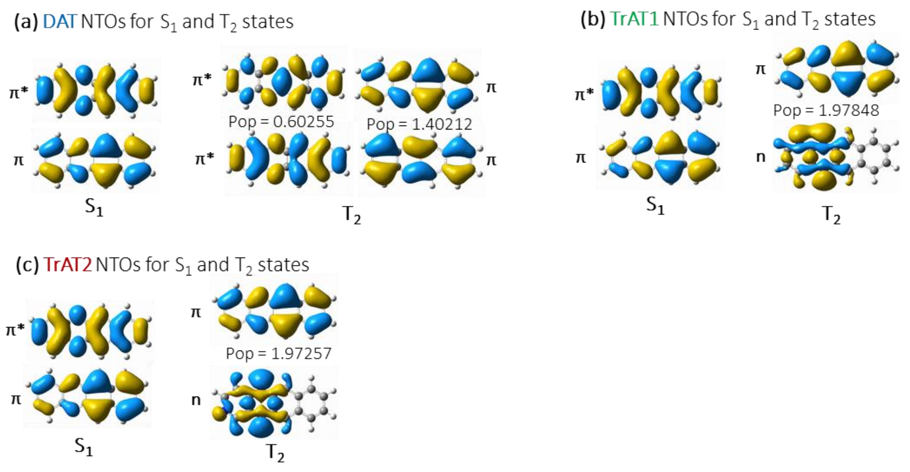

Figure S8. Natural Transition Orbitals (NTOs) for $S_0 \rightarrow S_1$ and $T_1 \rightarrow T_2$ transitions for (a) DAT, (b) TrAT1 and (c) TrAT2 calculated using TD-DFT. Populations represent the relative contributions to excited state, with orbital character identified as non-bonding (n), pi-bonding (π) or pi-antibonding (π*). Isovalue = 0.02 electrons/A^3.

Table S2. TD-DFT derived electronic excitations and oscillator strengths for **DAT**. Calculations were performed in Gaussian09 at the B3LYP 6-311G++(d,p) level.

**Compound**	**Symmetry**	**Transition**	**Excitation Energy eV/ nm**	**CI**	**Oscillator Strength**
	**Singlet**				
	$\mathbf{^1B_2}$	**60 -> 61**	**2.28/543**	**0.705**	**0.045**
	$^1B_1$	59 -> 61	2.87/432	0.703	0.001
	$\mathbf{^1A_1}$	**58 -> 61; 60 -> 63**	**3.38/366**	**0.630; 0.313**	**0.2979**
	$\mathbf{^1B_2}$	**57 -> 61**	**3.39/365**	**0.697**	**0.008**
	**Triplet**				
	$^3A_2$	55B -> 61B; 58B -> 60B	1.39/892	-0.106; 0.990	0.0000
	$\mathbf{^3A_1}$	**61A -> 62A; 59B -> 60B**	**1.53/812**	**-0.498; 0.861**	**0.0278**
	$^3B_2$	61A -> 63A; 57B -> 60B<br>59B -> 61B	1.85/670	0.148; 0.972<br>-0.115	0.0000
**DAT**	$\mathbf{^3A_1}$	**61A -> 62A; 61A -> 64A**<br>**56B -> 60B; 57B -> 61B**<br>**59B -> 60B**	**2.48/500**	**0.724; 0.167**<br>**-0.436; 0.235**<br>**0.425**	**0.1991**
	$^3B_1$	55B -> 60B; 58B -> 61B<br>58B -> 62B	2.49/499	-0.623; 0.748<br>-0.132	0.0008
	$^3B_2$	60A -> 62A; 61A -> 63A<br>54B -> 60B; 57B -> 60B	2.66/466	-0.157; 0.944<br>-0.127; -0.181	0.0002
	$\mathbf{^3A_1}$	**61A -> 62A; 61A -> 64A**<br>**53B -> 60B; 56B -> 60B**<br>**57B -> 61B; 59B -> 60B**	**2.71/457**	**0.361; -0.367**<br>**-0.125; 0.782**<br>**0.199; 0.198**	**0.1291**
	$^3B_2$	60A -> 62A; 61A -> 63A<br>54B -> 60B; 59B -> 61B	2.85/435	0.328; 0.146<br>-0.204; 0.876	0.0000

Table S3. TD-DFT derived electronic excitations and oscillator strengths for **TrAT1**. Calculations were performed in Gaussian09 at the B3LYP 6-311G++(d,p) level.

	**Singlet**				
	**^{1}A’**	**60 -> 61**	**2.22/558**	**0.705**	**0.0358**
	^{1}A’’	59 -> 61	2.60/476	0.703	0.0002
	**^{1}A’**	**58 -> 61; 60 -> 63**	**3.37/368**	**0.641;-0.288**	**0.3639**
	^{1}A’’	56 -> 61	3.73/332	0.697	0.0020
	**^{1}A’**	**57 -> 61; 60 -> 62**	**3.86/321**	**0.679; -0.154**	**0.0257**
	**^{1}A’**	**55 -> 61; 57 -> 61**<br>**60 -> 62**	**4.11/302**	**-0.145; 0.107**<br>**0.675**	**0.0267**
	**Triplet**				
	^{3}A’’	56B -> 60B; 59B -> 60B	1.16/1070	0.145; 0.981	0.0000
	^{3}A’	61A -> 62A; 57B -> 60B<br>58B -> 60B	1.76/704	-0.627; -0.126<br>0.761	0.0012
	^{3}A’	61A -> 62A; 61A -> 63A<br>57B -> 60B	1.84/672	-0.105; -0.144<br>0.964	0.0000
**TrAT1**	^{3}A’’	54B -> 60B; 56B -> 60B<br>56B -> 61B; 59B -> 60B<br>59B -> 61B	2.21/560	0.247; 0.866<br>-0.165; -0.135<br>-0.347	0.0004
	^{3}A’’	54B -> 60B; 56B -> 60B<br>59B -> 61B; 59B -> 62B	2.37/523	-0.348; 0.432<br>0.800; -0.135	0.0001
	**^{3}A’’**	**60A -> 63A; 61A -> 62A**<br>**61A -> 64A; 55B -> 60B**<br>**57B -> 61B; 58B -> 60B**	**2.62/474**	**-0.120; 0.689**<br>**0.121; -0.180**<br>**0.339; 0.575**	**0.3202**
	^{3}A˙	60A -> 62A; 61A -> 63A<br>53B -> 60B; 55B -> 60B<br>57B -> 60B	2.68/462	0.139; 0.938<br>0.163; -0.104<br>0.169	0.0005
	^{3}A˙	61A -> 62A; 61A -> 63A<br>61A -> 64A; 61A -> 65A<br>55B -> 60B; 58B -> 60B	2.69/460	0.154; 0.120<br>-0.414; 0.132<br>0.842; 0.165	0.0032

Table S4. TD-DFT derived electronic excitations and oscillator strengths for **TrAT2**. Calculations were performed in Gaussian09 at the B3LYP 6-311G++(d,p) level.

	**Singlet**				
	**^{1}A’**	**60 -> 61**	**2.19/656**	**0.705**	**0.0393**
	^{1}A’’	59 -> 61	2.73/454	0.698	0.0005
	**^{1}A’**	**58 -> 61; 60 -> 62<br>60 -> 63**	**3.33/372**	**0.657; -0.188; 0.164**	**0.2136**
	^{1}A’’	56 -> 61	3.38/366	0.694	0.0013
	**^{1}A’**	**57 -> 61; 60 -> 62**	**3.58/346**	**0.655; 0.23**	**0.0368**
	**Triplet**				
	^{3}A’’	57B -> 60B; 59B -> 60B	1.34/923	-0.272<br>0.952	0.0000
	**^{3}A’**	**61A -> 62A; 58B -> 60B**	**1.56/795**	**0.458<br>0.879**	**0.0285**
	^{3}A’	61A -> 62A; 61A -> 63A<br>56B -> 60B; 58B -> 61B	2.00/621	0.258; 0.157<br>0.934; -0.112	0.0007
**TrAT2**	^{3}A’’	57B -> 60B; 59B -> 60B	2.00/621	0.951; 0.277	0.0001
	^{3}A’’	54B -> 60B; 57B -> 61B<br>59B -> 61B; 59B -> 62B	2.45/506	0.545; -0.259<br>0.762; -0.119	0.0006
	**^{3}A’**	**61A -> 62A; 61A -> 64A<br>55B -> 60B; 56B -> 60B<br>56B -> 61B; 58B -> 60B<br>58B -> 61B**	**2.51/495**	**0.676; -0.135<br>0.464; -0.178<br>-0.163; -0.388<br>0.260**	**0.1626**
	**^{3}A’**	**61A -> 62A; 61A -> 63A<br>61A -> 64A; 55B -> 60B<br>56B -> 60B; 56B -> 61B<br>58B -> 60B; 58B -> 61B**	**2.65/467**	**0.298; 0.634<br>0.229; -0.587<br>-0.181; -0.101<br>-0.107; 0.171**	**0.0554**
	**^{3}A’**	**60A -> 62A; 61A -> 62A<br>61A -> 63A; 61A -> 64A<br>55B -> 60B; 56B -> 60B<br>58B -> 60B; 58B -> 61B**	**2.72/457**	**-0.192; -0.157<br>0.701; -0.241<br>0.505; -0.118<br>0.114; -0.258**	**0.0339**

Table S5. Reduced spin-orbit coupling matrix elements (SOCME) for **DAT**. Calculations performed in Orca v5.0.1 at the B3LYP/def-TZVP level.

Multiplicity & State		$\langle S_n \vert H_{SO} \vert T_x \rangle$	$\langle S_n \vert H_{SO} \vert T_y \rangle$	$\langle S_n \vert H_{SO} \vert T_z \rangle$
Singlet, S	Triplet, T			
0	0	0	0	0
0	1	0	0	0
0	2	0	0	0
0	3	-3.15	-3.09	10.87
0	4	0.02	-0.02	0
0	5	0	0	0
1	0	0.04	-0.03	0.01
1	1	0	0	0
1	2	0	0	0
1	3	0	0	0
1	4	0	0	0
1	5	0	0	0
2	0	0	0	0
2	1	2.27	2.22	-7.83
2	2	0	0	0
2	3	0	0	0
2	4	-0.4	-0.72	-0.32
2	5	0	0	0
3	0	0	0	0
3	1	0.01	-0.01	0
3	2	0	0	0
3	3	0.54	0.99	0.44
3	4	0	0	0
3	5	0	0	0

Table S6. Reduced spin-orbit coupling matrix elements (SOCME) for **TrAT1**. Calculations performed in Orca v5.0.1 at the B3LYP/def-TZVP level.

Multiplicity & State		$\langle S_n\|H_{SO}\|T_x\rangle$	$\langle S_n\|H_{SO}\|T_y\rangle$	$\langle S_n\|H_{SO}\|T_z\rangle$
Singlet, S	Triplet, T			
0	0	0	0	0
0	1	0	0	0
0	2	6.12	0.79	0
0	3	0	0	0.04
0	4	9.35	-1.04	0
0	5	0	0	0
1	0	7.29	-1.63	0
1	1	4.76	0.46	0
1	2	0	0	-2.9
1	3	-3.06	-0.78	0
1	4	0	0	0
1	5	-3.06	-0.78	0
2	0	0	0	-0.05
2	1	0	0	0
2	2	-2.44	-0.81	0
2	3	0	0	-0.01
2	4	-9.48	0.14	0
2	5	0	0	0
3	0	0	0	0.01
3	1	0.17	0.62	0
3	2	0	0	0
3	3	0	0	0
3	4	0	0	0
3	5	0	0	0

Table S7. Reduced spin-orbit coupling matrix elements (SOCME) for **TrAT2**. Calculations performed in Orca v5.0.1 at the B3LYP/def-TZVP level.

Multiplicity & State		$\langle S_n\|H_{SO}\|T_x\rangle$	$\langle S_n\|H_{SO}\|T_y\rangle$	$\langle S_n\|H_{SO}\|T_z\rangle$
**Singlet, S**	**Triplet, T**			
0	0	0	0	-0.02
0	1	0	0	0
0	2	-8.37	6.39	0
0	3	-4.33	-2.13	0
0	4	0	0	-0.03
0	5	0	0	-0.03
1	0	1.35	3.93	0
1	1	-5.92	4.1	0
1	2	0	0	0.56
1	3	0	0	-5.6
1	4	3.08	-0.92	0
1	5	-3.83	4.46	0
2	0	0	0	0.03
2	1	0	0	0
2	2	5.08	-3.78	0
2	3	1.75	2.26	0
2	4	0	0	0
2	5	0	0	0
3	0	-4.16	-11.73	0
3	1	-2.27	-1.8	0
3	2	0	0	5.54
3	3	0	0	0.4
3	4	0.39	-0.07	0
3	5	-1.18	-4.25	0

Table S8. TD-DFT calculated electron-nucleus hyperfine coupling for nitrogen atoms. Calculations were performed in Orca v5.0.1 software at the B3LYP def2-TZVP/C level. N1 & N2 occupy 5,12-positions and N3 occupies the 1- or 2- position for **TrAT1** and **TrAT2**, respectively.

	Hyperfine Coupling / MHz		
	**N1 (x, y, z)**	**N2 (x, y, z)**	**N3 (x, y, z)**
**DAT**	8.28, 8.19, 30.06	8.29, 8.18, 30.06	-
**TrAT1**	7.69, 7.90, 28.55	7.20, 7.42, 27.60	0.440, 0.637, 4.284
**TrAT2**	8.06, 7.94, 29.48	8.53, 8.42, 30.85	0.433, 0.463, 3.71

Simulation of Triplet Signals

The least squares fitting of X-band triplet signals were performed using the “esfit” function inside the EasySpin (v6.0.0) package for MATLAB 2022a, with data “as is” using the Nelder/Mead simplex algorithm. The following typical script was used to load the EPR data, perform a background correction, then establish a starting point for the fitting, and subsequently conduct the fit (updated for compatibility with EasySpin v6.0.0 dev.51):

```
% Load in the Data
[B,spc, param] = eprload(['file_location.DTA']);

% Data Conversion – separation of real and imaginary data sets
spc_real = real(spc);
spc_imag = imag(spc);

% Background Correction - removal of laser noise by subtracting a time trace of a field position without sample-dependent signal
spc_real_corr = spc_real(:, :) - spc_real(:, 1);
Bmag = B{1,2};

% Spin and experimental parameters
Sys = struct('S',1,'g',[2.001],'lw',[0.5 0.5], 'D',[1600 150]);
Sys.initState = {[0.4 0.5 0.6],'xyz'};

Exp = struct('mwFreq',param.MWFQ*10^-9,'Harmonic',0,'Temperature',[1 1 15]);
Exp.Range = [273 423];

% Fitting Parameters
Vary = struct('lw',[2 2],'g',[0.01],'D',[250 100]);
Vary.initState = {[1 1 1], 'xyz'};

% Least square fitting
esfit(spc_real_corr(50,:), @pepper, {Sys,Exp}, {Vary});
```

During the fit the minimum and maximum allowed values for “initState”, which determines the triplet sublevel populations, were set to 0 and 1, respectively. The final simulated values for then normalised to a total of 1 to obtain the reported triplet populations. Similarly, the minimum value for “lw” was set to zero to prevent non-physical negative values from arising.

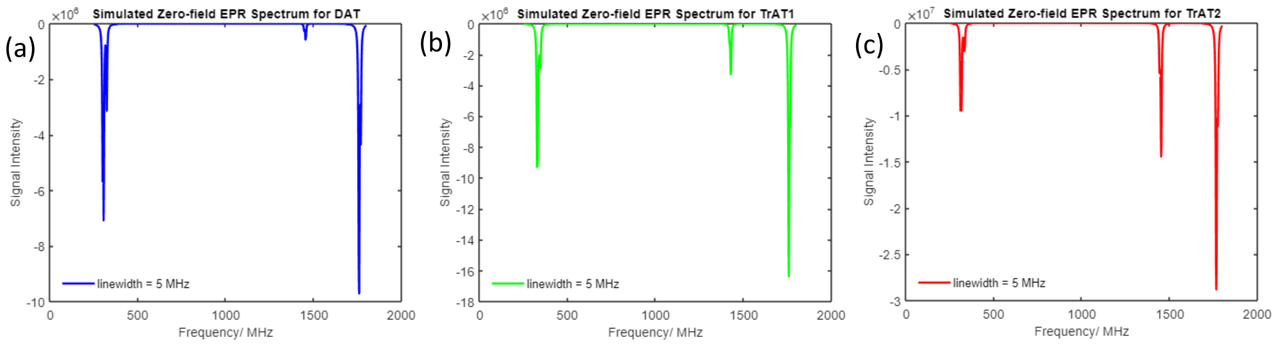

Figure S9: Simulated frequency swept zero-field EPR spectrum. Triplet populations were determined by fitting of X-band spectra while hyperfine splitting values were estimated using TD-DFT calculations at the B3LYP def-TZVP level, performed in Orca v5.0.1 software. The simulations were then performed in MATLAB 2022a with EasySpin v6.0.0.

Additional Zero-field EPR Spectroscopy

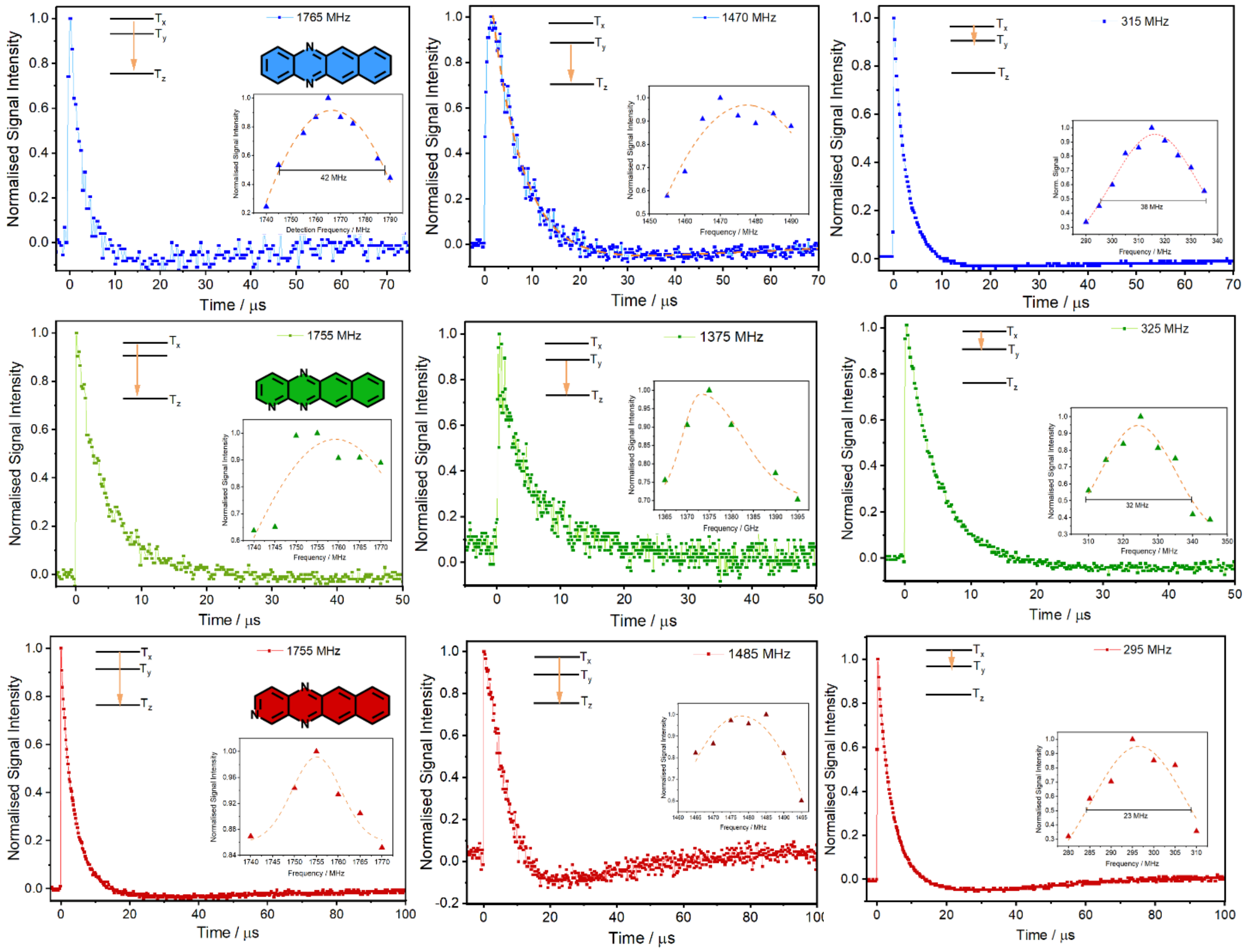

Figure S10. Individual zero-field time resolved EPR spectra for DAT:1-TNB (blue), TrAT1:1-TNB (green) and TrAT2:1-TNB (red) with inset frequency dependent signal intensity variations.

Q-boosting Maser Experiments & Dielectric Resonator Design

For maser experiments, samples were inserted into a cylindrical 12 mm (OD) x 4 mm (height) single crystal strontium titanate (STO) dielectric resonator with 6 mm (ID) bore. The resonator was supported above the base of an oxygen-free copper cavity by a low loss, crosslinked polystyrene support (**Figure S11a & b**). To precisely adjust the resonant frequency of the cavity, the lid height of the copper cavity was adjusted such that the $TE_{01\delta}$ mode was centred at the frequency of the $T_X \rightarrow T_Z$ transition for each sample.

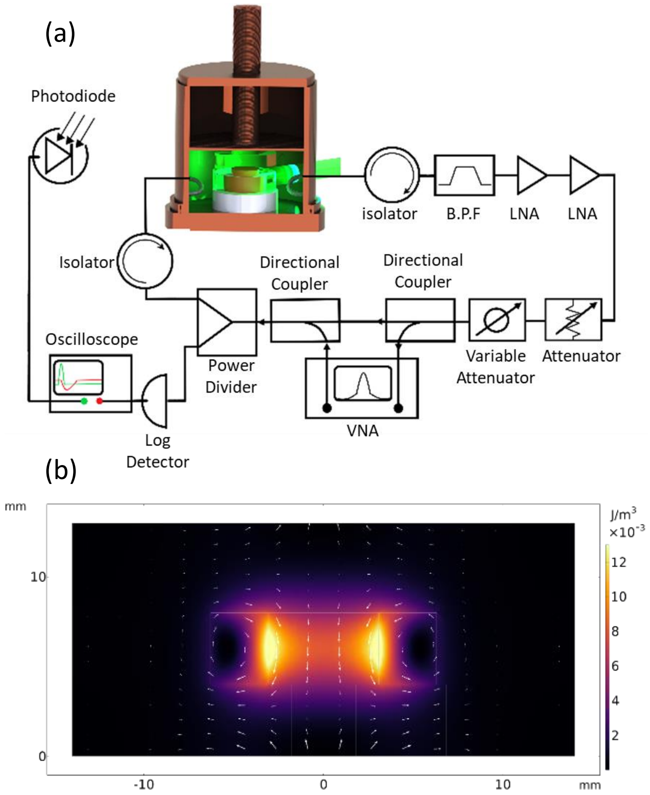

Figure S11: (a) Set up for Q-boosted maser experiments; (b) Plot of magnetic energy-density distribution showing the highest field density close to the walls of the 6 mm bore.

Q-boosted maser experiments were conducted on all samples to estimate the loaded cavity quality factor (Q_L) required to surpass the maser cooperativity threshold, previously described in ref [4] (**Figure S11a**). Briefly, an adjustable feedback loop consisting of variable attenuators and amplifiers was connected across the two inductive coupling loops that were inserted into the cavity. This permitted out-coupled power to be amplified in the feedback loop before being fed back into the cavity, thus compensating for radiative losses and artificially increasing the Q_L of the cavity, which was measured using a vector network analyser (VNA, HP 8753A). To optimise the effective Q_L, the gain and phase were also adjusted between subsequent experiments.

Prior to each measurement, the VNA was disconnected, and the photoexcitation was provided by an OPO (Litron Aurora II Integra, pulse length 5.5 ns, repetition rate 10 Hz, λ_{pump} 530 nm for **TrAT1**:1-TNB & **TrAT2**:1-TNB, 510 nm for **DAT**:1-TNB, pump power 40 mJ/pulse). The laser beam was aligned and focused through a hole in the copper cavity and illuminated the sample through the side of the STO resonator. Scattered light from the laser was detected using a photodiode (ThorLabs 201/579-7227) and used to trigger the oscilloscope (Keysight,

InfiniiVision DSOX6002A), which recorded the maser responses as measured by the log detector (AD8317).

References

(1) Horbury, M. D.; Baker, L. A.; Rodrigues, N. D. N.; Quan, W.-D.; Stavros, V. G. Photoisomerization of Ethyl Ferulate: A Solution Phase Transient Absorption Study. *Chem. Phys. Lett.* **2017**, *673*, 62–67. https://doi.org/10.1016/j.cplett.2017.02.004.

(2) Frisch, M. J.; Trucks, G. W.; Schlegel, H. B.; Scuseria, G. E.; Robb, M. A.; Cheeseman, J. R.; Scalmani, G.; Barone, V.; Mennucci, B.; Petersson, G. A.; Nakatsuji, H.; Caricato, M.; Li, X.; Hratchian, H. P.; Izmaylov, A. F.; J. Bloino, G. Z.; Sonnenberg, J. L.; Hada, M.; Ehara, M.; Toyota, K.; Fukuda, R.; Hasegawa, J.; Ishida, M.; Nakajima, T.; Honda, Y.; Kitao, O.; Nakai, H.; Vreven, T.; J. A. Montgomery, J.; Peralta, J. E.; Ogliaro, F.; Bearpark, M.; Heyd, J. J.; Brothers, E.; Kudin, K. N.; Staroverov, V. N.; Kobayashi, R.; Normand, J.; Raghavachari, K.; Rendell, A.; Burant, C. J.; Iyengar, S. S.; Tomasi, J.; Cossi, M.; Rega, N.; Millam, J. M.; Klene, M.; Knox, J. E.; Cross, J. B.; Bakken, V.; Adamo, C.; Jaramillo, J.; Gomperts, R.; Stratmann, R. E.; Yazyev, O.; Austin, A. J.; Cammi, R.; Pomelli, C.; Ochterski, J. W.; Martin, R. L.; Morokuma, K.; Zakrzewski, V. G.; Voth, G. A.; Salvador, P.; Dannenberg, J. J.; Dapprich, S.; Daniels, A. D.; Farkas, Ö.; Foresman, J. B.; Ortiz, J. V.; J. Cioslowski; Fox, D. J. *Gaussian 09*; Gaussian, Inc.: Wallingford CT, 2009.

(3) Tirado-Rives, J.; Jorgensen, W. L. Performance of B3LYP Density Functional Methods for a Large Set of Organic Molecules. *J. Chem. Theory Comput.* **2008**, *4* (2), 297–306. https://doi.org/10.1021/ct700248k.

(4) Ng, W.; Zhang, S.; Wu, H.; Nevjestic, I.; White, A. J. P.; Oxborrow, M. Exploring the Triplet Spin Dynamics of the Charge-Transfer Co-Crystal Phenazine/1,2,4,5-Tetracyanobenzene for Potential Use in Organic Maser Gain Media. *J. Phys. Chem. C* **2021**, *125* (27), 14718–14728. https://doi.org/10.1021/acs.jpcc.1c01654.
